# Spatial navigation questionnaires as a supportive diagnostic tool in early Alzheimer’s disease

**DOI:** 10.1016/j.isci.2024.109832

**Published:** 2024-04-26

**Authors:** Martina Laczó, Radka Svatkova, Ondrej Lerch, Lukas Martinkovic, Terezie Zuntychova, Zuzana Nedelska, Hana Horakova, Martin Vyhnalek, Jakub Hort, Jan Laczó

**Affiliations:** 1 Memory Clinic, Department of Neurology, Second Faculty of Medicine, Charles University and Motol University Hospital, Prague, Czechia

**Keywords:** Disease, Neuroscience, Clinical neuroscience

## Abstract

Impaired spatial navigation is early marker of Alzheimer’s disease (AD). We examined ability of self- and informant-reported navigation questionnaires to discriminate between clinically and biomarker-defined participants, and associations of questionnaires with navigation performance, regional brain atrophy, AD biomarkers, and biomarker status. 262 participants (cognitively normal, with subjective cognitive decline, amnestic mild cognitive impairment [aMCI], and mild dementia) and their informants completed three navigation questionnaires. Navigation performance, magnetic resonance imaging volume/thickness of AD-related brain regions, and AD biomarkers were measured. Informant-reported questionnaires distinguished between cognitively normal and impaired participants, and amyloid-β positive and negative aMCI. Lower scores were associated with worse navigation performance, greater atrophy in AD-related brain regions, and amyloid-β status. Self-reported questionnaire scores did not distinguish between the groups and were weakly associated with navigation performance. Other associations were not significant. Informant-reported navigation questionnaires may be a screening tool for early AD reflecting atrophy of AD-related brain regions and AD pathology.

## Highlights

• Informant-reported questionnaires identified participants with cognitive impairment
• Informant-reported questionnaires distinguished amyloid-β positive and negative aMCI
• Informant-reported questionnaires were associated with spatial navigation performance
• The questionnaires were also associated with AD-related brain atrophy and biomarkers

## Graphical abstract

**Figure:**
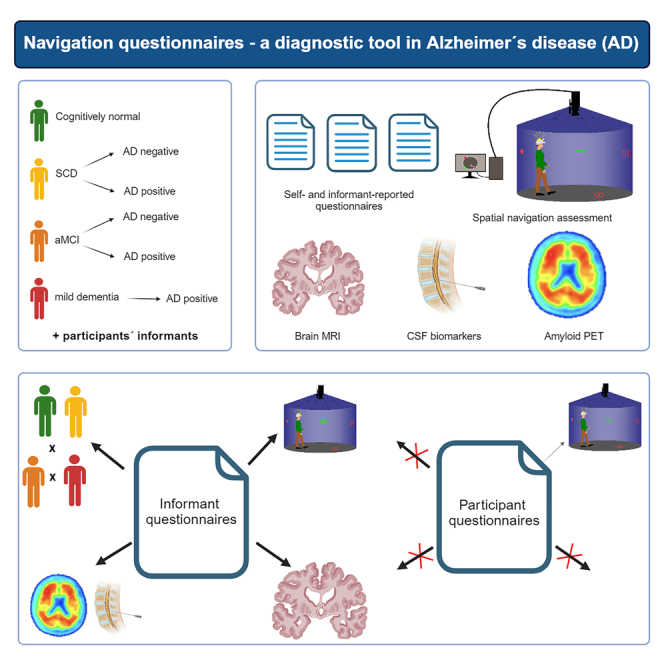

## Introduction

The aging population is leading to an increase in the number of people with dementia, making dementia a significant socio-economic burden.^1^ The most common cause of dementia is Alzheimer’s disease (AD) that may account for 60–80% of cases.^2^ AD follows a progressive course that ranges from an asymptomatic phase with amyloid-β and tau biomarkers suggestive of AD (preclinical AD) through mild cognitive impairment (MCI) and ultimately to dementia.^3^ Scientific advances in the development of disease-modifying treatments for AD have raised the urgent need for early and accurate diagnosis of AD.^4^ The development of cerebrospinal fluid (CSF) and positron emission tomography (PET) AD biomarkers has allowed early and accurate diagnosis through the detection of amyloid-β and tau pathology *in vivo*.^3^ However, their use is limited to specialized centers due to the invasiveness of CSF sampling and the high costs and limited availability of PET imaging.^5^ Emerging less invasive blood-based biomarkers have not yet been validated for clinical use.^6^ Therefore, it is of the utmost importance to find non-invasive and cost-effective markers of the disease that could be widely used as a screening tool to identify individuals at risk for AD. Spatial navigation impairment, which emerges very early in AD, has recently been suggested as a promising marker of the disease.^7^

Spatial navigation is a multifaceted cognitive process that allows us to move meaningfully in our environment and to find our way from one place to another. Based on the strategy used, it can be classified as egocentric (i.e., self-centered) and allocentric (i.e., world-centered) navigation.^8^ In egocentric navigation, spatial information about places and objects is encoded from the viewpoint of the navigator. In allocentric navigation, places and objects are encoded in relation to other visible objects independently of the navigator’s position and are stored in the form of a cognitive map. A large network of brain regions underlies our ability to navigate. Previous work suggests that the posterior parietal cortex^9^ and the precuneus^10^ are important for egocentric navigation, the hippocampus^11^^,^^12^ and the entorhinal cortex,^13^ especially their posterior subregions,^14^^,^^15^ as well as the basal forebrain^16^ are important for allocentric navigation, and the retrosplenial cortex is important for integration of egocentric and allocentric spatial information.^17^ Brain regions supporting spatial navigation, including the hippocampus, entorhinal cortex, basal forebrain, and parietal cortex, are the regions with early and progressive accumulation of tau and amyloid-β pathology, respectively.^18^^,^^19^ This could explain why individuals with AD are frequently disoriented in unfamiliar environments and, in later stages, may also be disoriented in familiar places.^20^ Spatial navigation deficits with impaired allocentric navigation may be detectable in preclinical AD,^21^ are more pronounced in amnestic MCI (aMCI), typically involving impairments in both allocentric and egocentric navigation,^22^^,^^23^ and are most extensive and severe in AD dementia.^23^^,^^24^ They have also been reported in individuals with subjective cognitive decline (SCD),^25^ i.e., those with self-perceived cognitive impairment but no objectively detectable cognitive deficits, who are thought to be at higher risk for preclinical AD.^26^ Recent studies have shown that spatial navigation deficits are more typical and prominent in aMCI individuals with positive compared to those with negative AD biomarkers^22^^,^^23^^,^^27^ and are associated with higher levels of AD pathology as measured by amyloid-β and phosphorylated tau (p-tau) in CSF.^21^^,^^23^^,^^28^

Therefore, spatial navigation deficits have a great potential to serve as early marker for AD. However, spatial navigation testing may be time consuming and not widely available, which may hinder its widespread use in clinical settings. Spatial navigation questionnaires can offer an opportunity to assess spatial navigation abilities in a clinically feasible and time-efficient manner, as questionnaire scores have been found to predict objective measures of these abilities quite reliably in young^29^^,^^30^ and older adults.^31^ Therefore, self- (-s) and informant- (-i) reported spatial navigation questionnaires may be readily available clinical screening tools for individuals at risk for AD. This is supported by the results of previous studies showing that self-reported spatial navigation deficits are related to AD pathology in cognitively normal (CN) older adults as measured by levels of amyloid-β in CSF^28^^,^^32^ and are common in memory clinic cohorts, including individuals with SCD, aMCI and AD dementia.^33^ Both self- and informant-reported spatial navigation questionnaires have been shown to identify individuals with AD dementia with episodes of getting lost,^34^ but only informant-reported questionnaires predicted getting lost episodes over a three-year period.^35^ It is worth noting that self-reported spatial navigation abilities may be influenced by anxiety and depressive symptoms.^33^^,^^36^ A recent study also showed that self-reported spatial navigation abilities may not predict objectively measured sense of location in the real world in individuals with aMCI and mild AD dementia.^37^ In addition, the extent of life-space mobility (i.e., the measure of movement extending from within one’s own home to movement beyond one’s own town or geographic region^38^ may interfere with reported spatial navigation abilities, as more frequent travel to more remote locations is more challenging.^38^^,^^39^ Although previous studies have suggested potential benefits and limitations of reported spatial navigation abilities in screening for AD, comparisons between self- and informant-reported spatial navigation questionnaires, and their associations with brain atrophy and biomarkers across the AD continuum have not been researched. There is also a lack of studies that have directly compared reported spatial navigation abilities with objective measures of egocentric and allocentric navigation strategies simultaneously in real-space and computerized environments using well-validated methods, such as the human analog of the Morris water maze (hMWM).^40^

To fill the knowledge gap, in the present study we comprehensively evaluated the diagnostic and clinical utility of three self- and informant-reported spatial navigation questionnaires in a memory clinic cohort from the Czech brain aging study (CBAS), to explore their potential as a screening tool for AD diagnosis. Specifically, we aimed to assess: (1) the ability of spatial navigation questionnaires to discriminate between CN older adults and participants from the memory clinic cohort defined by clinical criteria (i.e., with SCD, aMCI, and mild dementia) and stratified by AD biomarkers, (2) the associations of self- and informant-reported spatial navigation abilities with objective measures of real-space and computerized egocentric and allocentric spatial navigation performance in the hMWM, (3) the associations of atrophy in AD-related brain regions with reported navigation abilities and the role of objective spatial navigation measures in these associations, and (4) the associations of AD biomarker abnormalities and biomarker status with reported navigation abilities.

There is a lack of studies directly comparing self-reported and informant-reported spatial navigation abilities, but recent research on self-reported and informant-reported cognitive and memory complaints or decline has shown that informant-reported information is more consistent and reliable for diagnostic classification and disease prediction,^41^^,^^42^^,^^43^^,^^44^^,^^45^ and is more strongly associated with cognitive performance,^45^^,^^46^^,^^47^^,^^48^ AD-related structural brain changes,^45^^,^^46^^,^^47^^,^^49^ and AD biomarkers.^45^^,^^46^^,^^47^ Therefore, we hypothesized that spatial navigation abilities reported by informants would more reliably discriminate between clinical and AD biomarker groups than those reported by participants. Next, informant-reported versus self-reported spatial navigation abilities would show stronger associations with objectively measured egocentric and allocentric navigation strategies that are impaired in early AD.^40^ Finally, we hypothesized that, in particular, informant-reported poorer spatial navigation abilities would be associated with atrophy of the brain regions that show both early accumulation of AD pathology and association with spatial navigation, as well as with greater AD biomarker abnormalities, and biomarker positive status. Because there is a well-established relationship between spatial navigation performance and volume of brain regions affected early by AD pathology,^12^^,^^13^^,^^14^^,^^15^^,^^16^ we hypothesized that the associations between AD-related regional brain atrophy and reported poorer navigation abilities would be mediated by less accurate spatial navigation performance.

## Results

### Demographic, cognitive, spatial navigation, neuroimaging, and biomarker data

Demographic, cognitive, spatial navigation, and neuroimaging data are shown in Table 1. The SCD group was similar to the CN group in age, gender, education, and life-space mobility. The aMCI group was older and less educated than the SCD and CN groups, and had a lower proportion of women and more limited life-space mobility than the CN group. The mild dementia group had a lower proportion of women than the CN group, was less educated than the SCD group, and was older and had more limited life-space mobility than the other groups. The mild dementia group had more depressive symptoms than the CN group, while all groups had similar anxiety symptoms. The aMCI and mild dementia groups performed worse on most cognitive tests and on the real-space and computerized versions of the egocentric and allocentric navigation tasks than the SCD and CN groups, which were similar to each other. The aMCI and mild dementia groups had smaller volumes/thicknesses of most brain region measures than the SCD and CN groups. The SCD group had smaller left precuneus and right posterior parietal cortex thickness than the CN group. As shown in Table S1, here were essentially no differences between clinically defined participants with and without completed informant versions of the questionnaires. Correlations between spatial navigation performance, demographics, regional brain measures, AD biomarkers, and biomarker status in the memory clinic cohort are shown in Figure S1.

**Table 1:** Demographic, spatial navigation, cognitive and neuroimaging data

	CN (*n* = 41)	SCD (*n* = 76)	aMCI (*n* = 117)	Mild dementia (*n* = 28)	Total memory clinic cohort (*n* = 221)	F/Χ^2^	*P*
**Demographic characteristics**							

Age (years)	68.83 (6.07)	67.75 (7.55)	72.55 (7.47)^a^^b^	78.04 (7.50)^a^, ^b^, ^c^	71.59 (8.17)	16.61	<0.001
Women, n (%)	35 (85)	51 (67)	67 (57)	15 (54)	133 (60)	12.08	0.007
Education (years)	16.12 (2.58)	16.46 (2.85)	14.73 (2.73)^a^^b^	15.00 (3.13)^b^	15.36 (2.92)	7.00	0.002
LSA (score)	85.95 (14.36)	80.09 (17.60)	76.96 (19.26)^a^	68.11(26.97)^a^, ^b^, ^c^	76.94 (20.06)	5.18	0.002

**Spatial navigation performance**							

Real-space egocentric (distance error, cm)	25.47 (12.49)	24.99 (16.59)	44.52 (30.91)^a^^b^	70.80 (39.67)^a^, ^b^, ^c^	40.51 (31.17)	18.76	<0.001
Real-space allocentric (distance error, cm)	28.06 (12.86)	31.84 (18.65)	62.85 (36.34)^a^^b^	97.61 (31.62)^a^, ^b^, ^c^	55.51 (36.81)	28.19	<0.001
Computerized egocentric (distance error, pixels)	29.69 (19.63)	32.57 (19.91)	51.65 (28.38)^a^^b^	81.76 (36.26)^a^, ^b^, ^c^	48.75 (30.80)	27.06	<0.001
Computerized allocentric (distance error, pixels)	42.30 (23.26)	41.09 (21.76)	74.79 (32.04)^a^^b^	110.95 (28.36)^a^, ^b^, ^c^	67.58 (36.12)	37.87	<0.001

**Cognitive assessment**							

MMSE (score)	29.15 (1.01)	28.89 (1.27)	26.41 (2.79)^a^^b^	22.43 (2.74)^a^, ^b^, ^c^	26.76 (3.10)	73.36	<0.001
GDS-15 (score)	1.27 (1.52)	2.41 (2.37)	2.62 (2.37)	2.89 (2.90)^a^	2.58 (2.43)	3.86	0.010
BAI (score)	6.63 (5.84)	8.08 (5.87)	7.90 (7.72)	5.89 (5.51)	7.71 (6.89)	1.06	0.367
LM – immediate recall (score)	17.50 (2.85)	16.46 (3.77)	10.73 (4.54)^a^^b^	6.04 (3.60)^a^, ^b^, ^c^	12.13 (5.43)	75.57	<0.001
LM – delayed recall (score)	16.75 (3.49)	15.28 (3.57)	7.15 (5.69)^a^^b^	2.37 (4.08)^a^, ^b^, ^c^	9.37 (6.65)	97.10	<0.001
RAVLT 1–5 (score)	55.68 (6.48)	53.87 (7.76)	34.70 (8.10)^a^^b^	22.75 (9.20)^a^, ^b^, ^c^	41.80 (13.20)	146.80	<0.001
RAVLT 30 (score)	11.73 (2.00)	11.59 (2.30)	4.27 (3.06)^a^^b^	1.55 (2.70)^a^, ^b^, ^c^	7.13 (4.69)	160.92	<0.001
TMT A (seconds)	38.25 (10.75)	36.48 (9.45)	54.81 (22.28)^a^^b^	73.64 (37.66)^a^, ^b^, ^c^	50.79 (24.62)	29.77	<0.001
TMT B (seconds)	82.97 (32.65)	92.21 (36.92)	164.58 (82.64)^a^^b^	225.60 (84.03)^a^, ^b^, ^c^	147.07 (83.02)	43.45	<0.001
COWAT (score)	49.38 (9.54)	51.07 (10.39)	38.85 (11.82)^b^	34.74 (13.55)^a^^,^^b^	42.57 (13.14)	27.00	<0.001
ROCFT-C (score)	30.88 (3.33)	31.08 (3.07)	26.56 (5.78)^b^	24.63 (8.09)^a^^,^^b^	27.89 (5.87)	19.92	<0.001
ROCFT-R (score)	18.50 (6.84)	18.63 (5.81)	8.89 (6.26)^a^^b^	4.09 (3.34)^a^, ^b^, ^c^	11.66 (7.85)	71.60	<0.001
DSF (score)	9.35 (2.34)	9.54 (2.11)	8.48 (1.99)^a^^b^	8.07 (1.59)^a^^,^^b^	8.80 (2.06)	6.24	<0.001
DSB (score)	6.58 (2.15)	6.71 (1.81)	5.70 (1.84)^a^^b^	5.19 (1.59)^a^^,^^b^	5.99 (1.88)	7.57	<0.001
CDT (score)	15.35 (1.00)	14.92 (1.32)	14.09 (2.16)^a^^b^	12.70 (3.10)^a^, ^b^, ^c^	14.21 (2.16)	12.91	<0.001
SVF Animals (score)	26.98 (5.15)	27.05 (5.15)	18.88 (5.81)^a^^b^	14.33 (5.51)^a^, ^b^, ^c^	21.15 (7.15)	62.46	<0.001
SVF Vegetable (score)	14.48 (3.10)	13.92 (3.37)	9.61 (3.30)^a^^b^	7.11 (2.74)^a^, ^b^, ^c^	10.79 (4.05)	55.08	<0.001
BNT (no. of errors)	2.15 (2.62)	1.58 (2.34)	4.89 (3.87)^a^^b^	6.48 (3.08)^a^, ^b^, ^c^	3.94 (3.74)	26.43	<0.001
PST (seconds)	29.28 (6.64)	29.05 (7.71)	45.70 (37.55)^a^^b^	57.29 (27.52)^a^, ^b^, ^c^	41.37 (30.84)	11.50	<0.001

**Regional brain measures**^d^							

Hippocampus posterior right (volume, cm^3^)^e^	1.23 (0.16)	1.23 (0.13)	1.09 (0.22)^a^^,^^b^	0.87 (0.18)^a^, ^b^, ^c^	1.11 (0.22)	27.93	<0.001
Hippocampus posterior left (volume, cm^3^)^e^	1.29 (0.16)	1.30 (0.12)	1.14 (0.21)^a^^,^^b^	0.94 (0.20)^a^, ^b^, ^c^	1.17 (0.22)	29.64	<0.001
pmEC right (volume, cm^3^)^e^	0.35 (0.05)	0.35 (0.06)	0.33 (0.06)	0.31 (0.06)	0.34 (0.06)	2.95	0.033
pmEC left (volume, cm^3^)^e^	0.39 (0.05)	0.39 (0.06)	0.37 (0.07)^a^^,^^b^	0.35 (0.06)^a^^,^^b^	0.37 (0.06)	4.48	0.004
BF total (volume, cm^3^)^e^	0.65 (0.13)	0.60 (0.10)	0.54 (0.11)^a^^,^^b^	0.47 (0.10)^a^, ^b^, ^c^	0.56 (0.12)	17.19	<0.001
Precuneus right (thickness, mm)	2.32 (0.14)	2.24 (0.14)	2.19 (0.19)^a^^,^^b^	2.08 (0.18)^a^, ^b^, ^c^	2.19 (0.18)	11.81	<0.001
Precuneus left (thickness, mm)	2.30 (0.13)	2.20 (0.15)^a^	2.15 (0.19)^a^^,^^b^	2.05 (0.17)^a^, ^b^, ^c^	2.16 (0.18)	12.68	<0.001
Retrosplenial cortex right (thickness, mm)	2.26 (0.12)	2.20 (0.15)	2.17 (0.15)^a^	2.17 (0.16)^a^	2.18 (0.15)	4.22	0.006
Retrosplenial cortex left (thickness, mm)	2.29 (0.13)	2.23 (0.15)	2.19 (0.15)^a^	2.13 (0.17)^a^^,^^b^	2.20 (0.15)	7.40	<0.001
Posterior parietal cortex right (thickness, mm)	2.32 (0.12)	2.25 (0.15)^a^	2.18 (0.18)^a^^,^^b^	2.08 (0.18)^a^, ^b^, ^c^	2.19 (0.18)	14.17	<0.001
Posterior parietal cortex left (thickness, mm)	2.31 (0.11)	2.26 (0.15)	2.18 (0.17)^a^^,^^b^	2.11 (0.16)^a^, ^b^, ^c^	2.20 (0.17)	12.96	0.001

**Figure 1:**
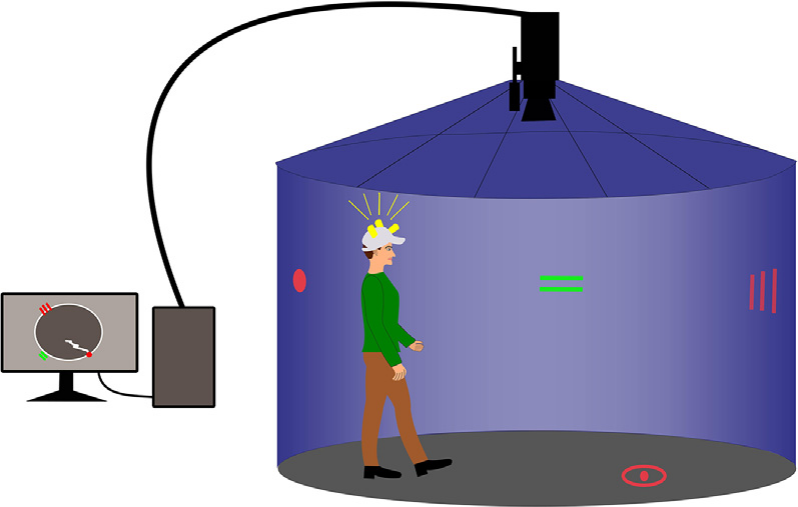
A real-space version of the human analog of the Morris water maze The participant is at the start position, at the red dot on the wall, there are two landmarks on the wall marked with green and red lines, and the goal is visible on the floor as a red circle. The participant wears a cap with LED diodes and his position is tracked by a camera mounted at the top of the arena.

**Figure 2:**
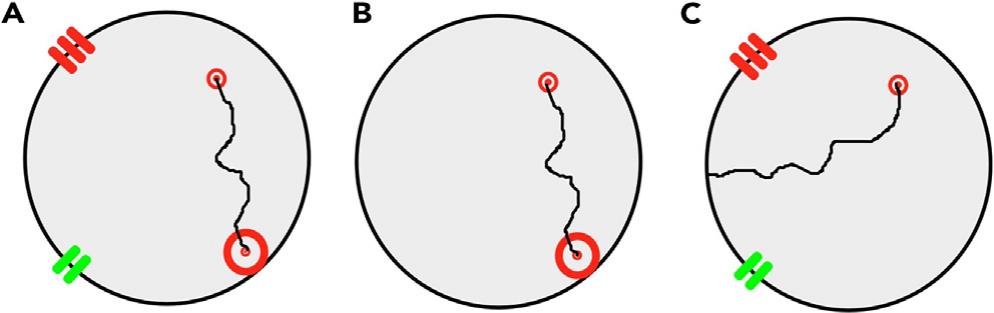
Three tasks in the human analog of the Morris water maze Computerized version of the human analog of the Morris water maze with three tasks: (A) allocentric-egocentric, (B) egocentric, and (C) allocentric. A large red circle represents the start position, red and green lines represent two landmarks, and a small red circle represents the goal. The black line shows the distance traveled by the participant from the start position to the goal.

Values are mean (SD) except for gender. *F*/Χ^2^ and *p* values refer to the main effect across all groups. Spatial navigation tasks are described in Figures 1 and 2.

**Key:** CN, cognitively normal; SCD, subjective cognitive decline; aMCI, amnestic mild cognitive impairment; LSA, Life-Space Assessment; MMSE, Mini-Mental State Examination; GDS-15, Geriatric Depression Scale 15-item version; BAI, Beck Anxiety Inventory; LM, Logical Memory; RAVLT, Rey Auditory Verbal Learning Test; RAVLT 1-5, trials 1 to 5 total; RAVLT 30, delayed word recall after 30 min; TMT A and B, Trail Making Tests A and B; COWAT, Controlled Oral Word Association Test (Czech version with letters N, K and P); ROCFT-C, Rey-Osterrieth Complex Figure Test – the Copy condition; ROCFT-R, Rey-Osterrieth Complex Figure Test – the Recall condition after 3 min; DSF, Digit Span Forward total score; DSB, Digit Span Backward total score; CDT, Clock Drawing Test – Cohen’s scoring; SVF, Semantic Verbal Fluency; BNT, Boston Naming Test (30-item version); PST, Prague Stroop Test – colors; pmEC, posteromedial entorhinal cortex; BF, basal forebrain.

^a-c^ Significant differences between the groups based on post-hoc analyses.

a Compared to the CN group.

b Compared to the SCD group.

c Compared to the aMCI group.

d Based on a sample with complete brain imaging data (*n* = 248) with CN (*n* = 39), SCD (*n* = 72), aMCI (*n* = 113) and mild dementia (*n* = 24) participants.

e Normalized to estimated total intracranial volume.

Biomarker, demographic, cognitive, spatial navigation, and neuroimaging data of the memory clinic cohort with CSF biomarkers and amyloid PET stratified by amyloid-β status are shown in Table 2. Table 3 shows the categorization of participants with biomarker data into eight AT(N) biomarker profiles. Stratification by amyloid-β status identified 70 A-negative and 67 A-positive participants. There were no differences in cognitive and spatial navigation performance, volumes/thicknesses of AD-related brain regions, and AD biomarkers between the amyloid-β negative and positive SCD groups except for the CSF amyloid-β_1-42_ levels and amyloid PET positivity. The amyloid-β negative aMCI group had better global cognition and memory, more accurate spatial navigation performance on the real-space and computerized versions of the egocentric and allocentric tasks, greater volume of the right posteromedial entorhinal cortex and thickness of the left precuneus, higher CSF amyloid-β_1-42_ levels, and did not have any amyloid PET positive participant compared to the amyloid-β positive aMCI group. As shown in Table S2, there were essentially no differences between biomarker-defined participants with and without completed informant versions of the questionnaires.

**Table 2:** Characteristics of the memory clinic cohort with biomarkers stratified by amyloid-β status

	SCD amyloid negative (*n* = 35)	SCD amyloid positive (*n* = 9)	aMCI amyloid negative (*n* = 35)	aMCI amyloid positive (*n* = 46)	Mild dementia amyloid positive (*n* = 12)	Total memory clinic cohort (*n* = 137)	F/Χ^2^	*P*
**Demographic characteristics**								

Age (years)	65.86 (7.37)	67.33 (4.66)	71.11 (8.53)^a^	72.85 (6.28)^a^	74.58 (8.14)^a^	70.41 (7.82)	6.18	<0.001
Women, n (%)	25 (71)	5 (56)	21 (60)	29 (63)	6 (50)	86 (63)	2.28	0.685
Education (years)	16.63 (2.91)	18.00 (4.00)	14.29 (2.35)^a^^,^^b^	15.07 (2.95)^a^^,^^b^	14.67 (2.71)^b^	15.42 (3.02)	5.18	<0.001
LSA (score)	80.97 (16.86)	90.67 (15.10)	78.64 (19.85)	78.61 (19.81)	72.73 (28.53)	79.55 (19.71)	1.14	0.341

**Spatial navigation performance**								

Real-space egocentric (distance error, cm)	23.37 (15.73)	21.03 (6.42)	34.53 (24.48)^a^	51.72 (33.64) a, b, c	86.22 (35.81)^a^, ^b^, ^c^, ^d^	40.72 (31.82)	14.06	<0.001
Real-space allocentric (distance error, cm)	29.76 (13.4)	23.74 (4.37)	52.86 (33.88)^a^	75.71 (37.24) a, b, c	102.85 (29.90)^a^, ^b^, ^c^, ^d^	56.41 (37.69)	13.61	<0.001
Computerized egocentric (distance error, pixels)	30.98 (19.44)	22.72 (8.64)	39.62 (23.05)^a^	64.17 (29.09) a, b, c	94.40 (39.49)^a^, ^b^, ^c^, ^d^	49.35 (32.39)	30.57	<0.001
Computerized allocentric (distance error, pixels)	36.71 (17.31)	31.13 (8.53)	66.51 (27.35)^a^^,^^b^	86.46 (31.73) a, b, c	110.90 (32.50) a, b, c	67.16 (36.11)	23.07	<0.001

**Cognitive assessment**								

MMSE (score)	28.89 (1.26)	28.44 (1.24)	27.29 (2.26)^a^	25.28 (2.70) a, b, c	23.67 (1.83)^a^, ^b^, ^c^, ^d^	26.78 (2.73)	22.36	<0.001
GDS-15 (score)	2.20 (2.10)	1.89 (1.36)	2.49 (2.66)	2.28 (1.85)	2.75 (2.70)	2.33 (2.18)	0.280	0.891
BAI (score)	7.69 (5.36)	5.78 (4.32)	6.31 (5.72)	7.78 (8.71)	5.92 (5.68)	7.09 (6.69)	0.481	0.750
LM – immediate recall (score)	16.34 (3.83)	17.44 (3.75)	13.34 (3.71)^a^^,^^b^	8.04 (3.45) a, b, c	7.00 (4.63) a, b, c	12.04 (5.33)	35.85	<0.001
LM – delayed recall (score)	15.20 (3.80)	16.11 (3.18)	10.51 (4.83)^a^^,^^b^	3.61 (4.08) a, b, c	3.67 (5.58) a, b, c	9.16 (6.65)	47.80	<0.001
RAVLT 1–5 (score)	53.69 (7.68)	57.11 (8.07)	37.00 (9.66)^a^^,^^b^	32.69 (6.77) a, b, c	27.33 (2.66) a, b, c	41.67 (12.76)	47.51	<0.001
RAVLT 30 (score)	11.63 (2.46)	12.67 (1.41)	5.29 (3.07)^a^^,^^b^	3.49 (3.10) a, b, c	2.17 (3.49) a, b, c	7.03 (4.72)	52.93	<0.001
TMT A (seconds)	32.81 (6.02)	37.39 (8.50)	56.05 (25.57)^a^^,^^b^	56.25 (22.60)^a^^,^^b^	77.60 (41.76)^a^, ^b^, ^c^, ^d^	50.84 (25.74)	11.91	<0.001
TMT B (seconds)	86.48 (29.44)	76.88 (15.77)	161.15 (77.90)^a^^,^^b^	173.88 (90.46)^a^^,^^b^	219.97 (83.61) a, b, c	145.96 (83.99)	13.36	<0.001
COWAT (score)	51.63 (9.49)	57.67 (11.60)	36.43 (13.47)^a^^,^^b^	41.96 (10.97) a, b, c	36.17 (11.16)^a^^,^^b^	43.54 (13.21)	12.79	<0.001
ROCFT-C (score)	30.76 (3.02)	33.33 (2.29)	28.13 (4.84)^a^^,^^b^	25.78 (5.85)^a^^,^^b^	22.08 (8.90)^a^, ^b^, ^c^, ^d^	27.83 (5.89)	10.81	<0.001
ROCFT-R (score)	19.03 (5.47)	22.22 (5.73)	10.96 (5.69)^a^^,^^b^	6.61 (5.72) a, b, c	3.21 (3.17)^a^, ^b^, ^c^	11.62 (8.10)	41.23	<0.001
DSF (score)	9.54 (2.42)	10.11 (2.57)	8.20 (1.84)	8.87 (2.19)	8.42 (1.51)	8.91 (2.19)	2.59	0.040
DSB (score)	7.03 (1.82)	7.11 (1.83)	5.74 (1.72)^a^	5.67 (1.93)^a^	5.33 (1.16)^a^	6.10 (1.88)	4.62	0.002
CDT (score)	14.71 (1.38)	15.11 (1.36)	14.71 (2.20)	13.41 (2.06) a, b, c	11.92 (2.64)^a^, ^b^, ^c^, ^d^	14.06 (2.15)	7.35	<0.001
SVF Animals (score)	27.80 (4.79)	28.89 (4.76)	20.57 (6.20)^a^^,^^b^	18.37 (4.60)^a^^,^^b^	14.08 (5.25)^a^, ^b^, ^c^, ^d^	21.66 (6.94)	28.26	<0.001
SVF Vegetable (score)	14.03 (3.02)	15.89 (2.89)	10.20 (3.62)^a^^,^^b^	9.28 (2.69)^a^^,^^b^	7.08 (2.35)^a^, ^b^, ^c^, ^d^	10.97 (3.92)	23.96	<0.001
BNT (no. of errors)	1.17 (1.47)	0.67 (0.87)	4.23 (3.36)^a^^,^^b^	4.91 (3.46)^a^^,^^b^	5.83 (1.99)^a^^,^^b^	3.58 (3.30)	13.78	<0.001
PST (seconds)	29.03 (6.80)	25.15 (4.59)	43.51 (46.49)	46.39 (17.50)^a^	62.61 (27.80)^a^^,^^b^	41.24 (28.69)	4.88	0.001

**Regional brain measures**^e^								

Hippocampus posterior right (volume, cm^3^)^f^	1.22 (0.12)	1.32 (0.13)	1.10 (0.26)^a^^,^^b^	1.06 (0.20)^a^^,^^b^	0.86 (0.10)^a^, ^b^, ^c^, ^d^	1.12 (0.22)	8.80	<0.001
Hippocampus posterior left (volume, cm^3^)^f^	1.29 (0.12)	1.37 (0.12)	1.18 (0.24)^a^^,^^b^	1.11 (0.20)^a^^,^^b^	0.90 (0.12)^a^, ^b^, ^c^, ^d^	1.18 (0.21)	10.85	<0.001
pmEC right (volume, cm^3^)^f^	0.35 (0.06)	0.38 (0.06)	0.36 (0.06)	0.31 (0.06)^a^, ^b^, ^c^	0.29 (0.03)^a^, ^b^, ^c^	0.34 (0.06)	5.67	<0.001
pmEC left (volume, cm^3^)^f^	0.38 (0.05)	0.42 (0.06)	0.38 (0.07)	0.35 (0.07)^b^	0.32 (0.05)^b^	0.37 (0.07)	3.27	0.014
BF total (volume, cm^3^)^f^	0.60 (0.09)	0.62 (0.09)	0.54 (0.13)^a^	0.53 (0.10)^a^	0.44 (0.10)^a^, ^b^, ^c^	0.55 (0.11)	5.28	<0.001
Precuneus right (thickness, mm)	2.25 (0.12)	2.26 (0.10)	2.18 (0.22)	2.16 (0.18)	2.03 (0.17)^a^, ^b^	2.18 (0.18)	3.57	0.009
Precuneus left (thickness, mm)	2.22 (0.14)	2.22 (0.12)	2.18 (0.21)	2.09 (0.14)^a^^,^^c^	1.95 (0.18)^a^, ^b^, ^c^, ^d^	2.15 (0.18)	6.36	<0.001
Retrosplenial cortex right (thickness, mm)	2.21 (0.13)	2.23 (0.10)	2.20 (0.18)	2.15 (0.12)	2.09 (0.14)	2.18 (0.14)	2.10	0.085
Retrosplenial cortex left (thickness, mm)	2.24 (0.14)	2.27 (0.07)	2.19 (0.20)	2.18 (0.14)	2.10 (0.15)	2.20 (0.16)	2.14	0.080
Posterior parietal cortex right (thickness, mm)	2.26 (0.13)	2.28 (0.15)	2.21 (0.22)	2.13 (0.17)^a^^,^^b^	2.05 (0.18)^a^, ^b^, ^c^	2.19 (0.18)	4.82	0.001
Posterior parietal cortex left (thickness, mm)	2.26 (0.13)	2.32 (0.13)	2.20 (0.18)	2.15 (0.17)^a^^,^^b^	2.07 (0.14)^a^^,^^b^	2.20 (0.17)	5.54	<0.001

**Biomarkers**								

CSF amyloid-β_1-42_ (pg/mL)^g^	998.33 (323.04)	540.40 (251.57)^a^	1106.14 (341.58)	467.86 (97.71)^a^^,^^c^	465.53 (103.07)^a^^,^^c^	691.51 (363.56)	3.49	0.036
CSF p-tau_181_ (pg/mL)^g^	36.31 (16.82)	72.73 (37.70)	69.25 (75.91)	159.97 (224.69)	107.06 (39.85)	112.31 (162.60)	4.55	0.014
CSF total tau (pg/mL)^g^	253.90 (114.00)	414.70 (175.77)	360.83 (253.27)	733.73 (522.90)	820.21 (642.95)	582.62 (482.77)	4.42	0.016
Amyloid PET positive, n (%)^h^	0/30 (0)	(8/8 (100)	0/27 (0)	29/29 (100)	6/6 (100)	43/100 (43)	17.19	<0.001

Values are mean (SD) except for gender and amyloid PET positivity. *F*/Χ^2^ and *p* values refer to the main effect across all groups. Spatial navigation tasks are described in Figures 1 and 2.

**Key:** SCD, subjective cognitive decline; aMCI, amnestic mild cognitive impairment; LSA, Life-Space Assessment; MMSE, Mini-Mental State Examination; GDS-15, Geriatric Depression Scale 15-item version; BAI, Beck Anxiety Inventory; LM, Logical Memory; RAVLT, Rey Auditory Verbal Learning Test; RAVLT 1–5, trials 1 to 5 total; RAVLT 30, delayed word recall after 30 min; TMT A and B, Trail Making Tests A and B; COWAT, Controlled Oral Word Association Test (Czech version with letters N, K and P); ROCFT-C, Rey-Osterrieth Complex Figure Test—the Copy condition; ROCFT-R, Rey-Osterrieth Complex Figure Test—the Recall condition after 3 min; DSF, Digit Span Forward total score; DSB, Digit Span Backward total score; CDT, Clock Drawing Test—Cohen’s scoring; SVF, Semantic Verbal Fluency; BNT, Boston Naming Test (30-item version); PST, Prague Stroop Test—colors; pmEC, posteromedial entorhinal cortex; BF, basal forebrain; CSF, cerebrospinal fluid.

^a-d^ Significant differences between the groups based on post-hoc analyses.

a Compared to the SCD amyloid negative group.

b Compared to the SCD amyloid positive group.

c Compared to the aMCI amyloid negative group.

d Compared to the aMCI amyloid positive group.

e Based on a sample with complete brain imaging data (*n* = 129) with SCD amyloid negative (*n* = 33), SCD amyloid positive (*n* = 9), aMCI amyloid negative (*n* = 33), aMCI amyloid positive (*n* = 45) and mild dementia amyloid positive (*n* = 9) participants.

f Normalized to estimated total intracranial volume.

g Based on a sample with CSF data (*n* = 66) with SCD amyloid negative (*n* = 7), SCD amyloid positive (*n* = 3), aMCI amyloid negative (*n* = 17), aMCI amyloid positive (*n* = 30) and mild dementia amyloid positive (*n* = 9) participants.

h Based on a sample with amyloid PET data (*n* = 100) with SCD (*n* = 38), aMCI (*n* = 56) and mild dementia amyloid positive (*n* = 6) participants.

**Table 3:** Categorization of participants from the memory clinic cohort with biomarker data into eight AT(N) biomarker profiles

AT(N) biomarker profiles	Number of participants
A-T-(N)-	14
A + T-(N)-	3
A + T-(N)+	0
A + T+(N)-	3
A + T+(N)+	35
A-T+(N)-	6
A-T-(N)+	2
A-T+(N)+	8

**Key**: A, amyloid; T, tau; N, neurodegeneration.

### Reported spatial navigation abilities

**Figure 3:**
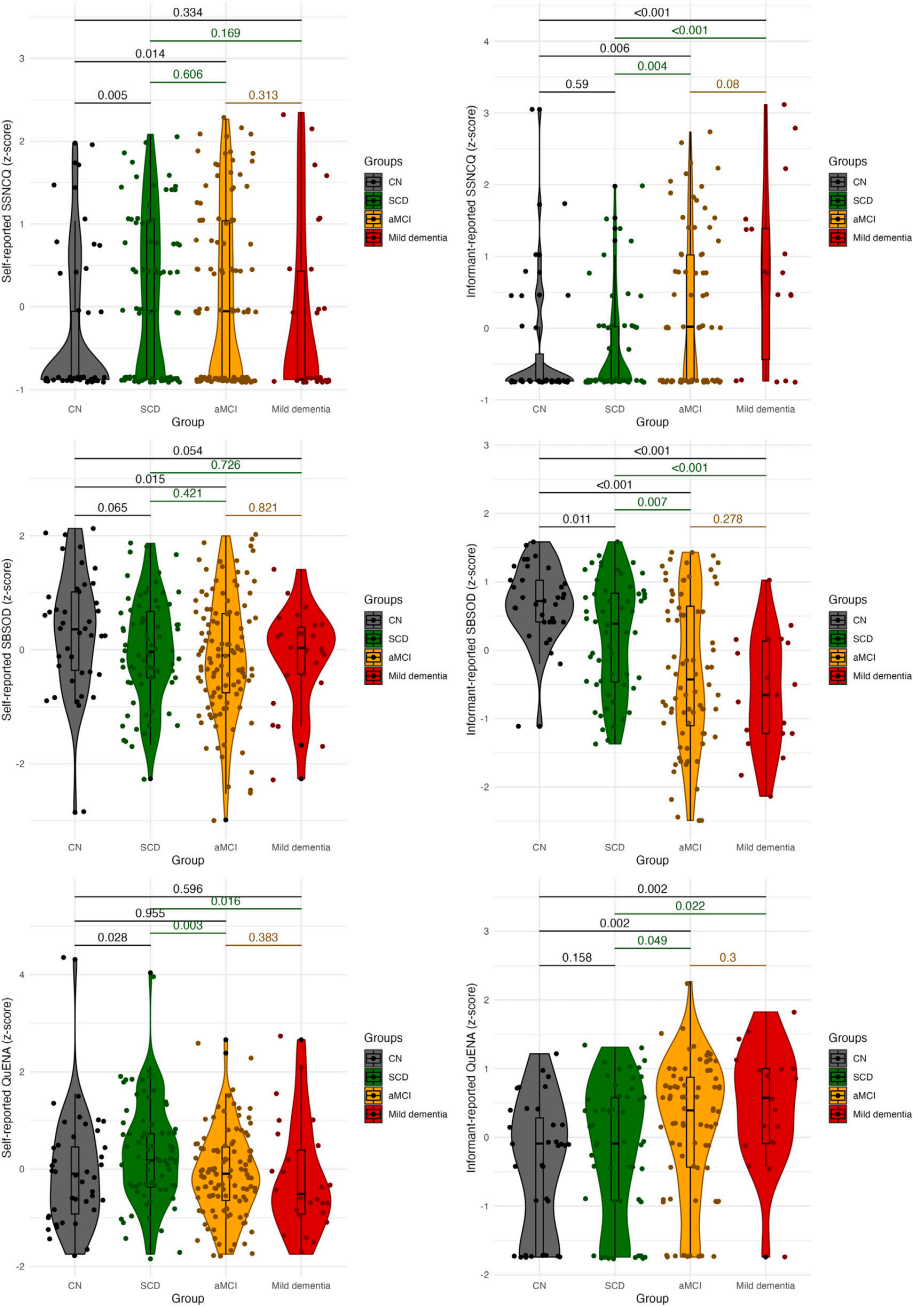
Differences between the clinical groups in self- and informant-reported spatial navigation questionnaires Key: CN, cognitively normal; SCD, subjective cognitive decline; aMCI, amnestic mild cognitive impairment; SSNCQ, Subjective Spatial Navigation Complaints Questionnaire; SBSOD, Santa Barbara Sense of Direction Scale; QuENA, Questionnaire on Everyday Navigational Ability.

The internal consistency was high for the self-reported questionnaires (Cronbach’s alpha = 0.81, 0.84, and 0.81 for the Subjective Spatial Navigation Complaints Questionnaire [SSNCQ-s], Santa Barbara Sense of Direction Scale [SBSOD-s] and Questionnaire on Everyday Navigational Ability [QuENA-s], respectively) and very high for the informant-reported questionnaires (Cronbach’s alpha = 0.92, 0.90, and 0.90 for the SSNCQ-i, SBSOD-i and QuENA-i, respectively). As shown in Figure 3 and Table 4, all informant-reported questionnaires distinguished the aMCI and mild dementia groups from the CN group, with the former two groups were reported to have poorer spatial navigation abilities. In addition, other between-group differences were found on the various informant-reported questionnaires. The SSNCQ-i distinguished the aMCI group from the SCD group, and the mild dementia group from all other groups. The SBSOD-i distinguished the aMCI and mild dementia groups from the SCD group, and the SCD group with reported poorer spatial navigation abilities from the CN group. The QuENA-i distinguished the mild dementia group from the SCD group. No significant differences were observed between the groups on self-reported questionnaires of spatial navigation abilities. The results of the receiver operating characteristic (ROC) analysis showing the discriminative ability of each questionnaire are presented in Table 5. Informant-reported questionnaires discriminated the aMCI and mild dementia groups from the CN group with areas under the curves (AUCs) ≥0.65 and ≥0.77, respectively, and the aMCI (excluding the QuENA-i) and mild dementia groups from the SCD group with AUCs ≥0.63 and ≥0.69, respectively. The SBSOD-i also discriminated the SCD group from the CN group with AUC of 0.66.

**Table 4:** Reported spatial navigation abilities

	CN (*n* = 41)	SCD (*n* = 76)	aMCI (*n* = 117)	Mild dementia (*n* = 28)	F	*P*
Self-reported SSNCQ (score)	1.12 (2.27)	2.22 (2.70)	2.35 (3.28)	2.04 (3.76)	2.77	0.042
Informant-reported SSNCQ (score)	1.56 (5.39)	0.91 (2.05)	3.01 (4.82)^a^^,^^b^	6.06 (9.00)^a^, ^b^, ^c^	7.92	<0.001
Self-reported SBSOD (score)	5.00 (1.04)	4.64 (0.93)	4.52 (1.08)	4.53 (0.86)	2.57	0.055
Informant-reported SBSOD (score)	5.83 (0.71)	5.20 (1.07)^a^	4.51 (1.44)^a^^,^^b^	4.10 (1.11)^a^^,^^b^	12.49	<0.001
Self-reported QuENA (score)	6.05 (4.08)	7.49 (3.70)	5.87 (3.14)	5.65 (4.10)	2.26	0.082
Informant-reported QuENA (score)	3.03 (2.94)	4.08 (3.40)	5.63 (4.58)^a^	6.95 (4.93)^a^^,^^b^	4.28	0.006

Values are mean (SD). *F* and *p* values refer to the main effect across all groups. The analysis was controlled for age, gender, education, and life-space mobility.

**Key**: CN, cognitively normal; SCD, subjective cognitive decline; aMCI, amnestic mild cognitive impairment; GDS-15, Geriatric Depression Scale 15-item version; BAI, Beck Anxiety Inventory; SSNCQ, Subjective Spatial Navigation Complaints Questionnaire; SBSOD, Santa Barbara Sense of Direction Scale; QuENA, Questionnaire on Everyday Navigational Ability.

^a-c^ Significant differences between the groups based on post-hoc analyses.

a Compared to the CN group.

b Compared to the SCD group.

c Compared to the aMCI group.

**Table 5:** ROC analysis of reported spatial navigation abilities

	Self-reported SSNCQ			Informant-reported SSNCQ			Self-reported SBSOD			Informant-reported SBSOD			Self-reported QuENA			Informant-reported QuENA		
	AUC	p	95% Cl	AUC	p	95% Cl	AUC	p	95% Cl	AUC	P	95% Cl	AUC	p	95% Cl	AUC	p	95% Cl
CN vs. SCD	0.646	0.010	0.541–0.750	0.534	0.594	0.410–0.658	0.604	0.065	0.495–0.713	0.659	0.011	0.548–0.771	0.624	0.028	0.515–0.732	0.591	0.147	0.472–0.711
CN vs. MCI	0.616	0.028	0.519–0.713	0.649	0.012	0.542–0.756	0.629	0.014	0.532–0.727	0.756	<0.001	0.667–0.844	0.501	0.992	0.391–0.610	0.684	0.002	0.581–0.787
CN vs. mild dementia	0.536	0.625	0.392–0.679	0.766	0.002	0.620–0.912	0.641	0.054	0.509–0.773	0.914	<0.001	0.828–1.000	0.461	0.593	0.317–0.605	0.767	0.002	0.630–0.904
SCD vs. MCI	0.477	0.594	0.392–0.561	0.630	0.012	0.535–0.725	0.535	0.421	0.451–0.619	0.638	0.007	0.544–0.731	0.367	0.002	0.285–0.450	0.598	0.058	0.499–0.696
SCD vs. mild dementia	0.393	0.105	0.263–0.522	0.761	0.001	0.616–0.906	0.523	0.724	0.400–0.647	0.764	0.001	0.644–0.884	0.341	0.016	0.209–0.473	0.685	0.019	0.542–0.829
MCI vs. mild dementia	0.422	0.217	0.300–0.544	0.631	0.085	0.486–0.776	0.486	0.819	0.373–0.598	0.581	0.276	0.453–0.709	0.447	0.403	0.314–0.581	0.592	0.226	0.445–0.739

**Key**: CN, cognitively normal; SCD, subjective cognitive decline; aMCI, amnestic mild cognitive impairment; AUC, area under the curve; 95% CI, 95% confidence interval; SSNCQ, Subjective Spatial Navigation Complaints Questionnaire; SBSOD, Santa Barbara Sense of Direction Scale; Questionnaire on Everyday Navigational Ability, QuENA.

As shown in Table 6, informant-reported navigation abilities, as measured by the SSNCQ-i and SBSOD-i, distinguished the amyloid-β positive aMCI group from the amyloid-β negative aMCI, CN, and both SCD groups. These differences were also observed in the amyloid-β positive mild dementia group. Informant-reported navigation abilities, as measured by the QuENA-i, distinguished the amyloid-β positive aMCI group from the CN group. Other between-group differences were not significant on these questionnaires. No significant differences were observed between the groups in self-reported navigation abilities, except for the SBSOD-s, which distinguished the amyloid-β positive aMCI group from the CN group. The results of the ROC analysis are presented in Table 7. Informant-reported questionnaires discriminated the amyloid-β positive aMCI group from the amyloid-β negative aMCI (excluding the QuENA-i) and CN groups and from the SCD negative and SCD positive (excluding the QuENA-i) groups with AUCs of 0.70, ≥0.73, ≥0.73, and ≥0.75, respectively, and the amyloid-β positive mild dementia group from the CN and amyloid-β negative SCD (excluding the QuENA-i) groups with AUCs ≥0.74 and ≥0.78, respectively. Among self-reported questionnaires, the SSNCQ-s and SBSOD-s discriminated the amyloid-β positive aMCI group from the CN group with AUCs ≥0.66.

**Table 6:** Reported spatial navigation abilities in the memory clinic cohort with biomarkers stratified by amyloid-β status

	CN (*n* = 41)	SCD amyloid negative (*n* = 35)	SCD amyloid positive (*n* = 9)	aMCI amyloid negative (*n* = 35)	aMCI amyloid positive (*n* = 46)	Mild dementia amyloid positive (*n* = 12)	F	*P*
Self-reported SSNCQ (score)	1.12 (2.27)	1.57 (2.05)	2.33 (1.94)	1.74 (2.51)	2.30 (2.96)	2.25 (3.75)	1.638	0.153
Informant-reported SSNCQ (score)	1.56 (5.39)	0.58 (2.20)	0.50 (1.12)	1.47 (3.06)	4.67 (5.87)^a^, ^b^, ^c^, ^d^	5.44 (7.50)^a^, ^b^, ^c^, ^d^	6.132	<0.001
Self-reported SBSOD (score)	5.00 (1.04)	4.75 (0.95)	4.89 (0.59)	4.76 (1.22)	4.22 (1.10)^a^	4.75 (0.72)	2.995	0.013
Informant-reported SBSOD (score)	5.83 (0.71)	5.29 (0.97)	5.70 (0.78)	4.98 (1.51)	3.95 (1.35)^a^, ^b^, ^c^, ^d^	3.80 (1.07)^a^, ^b^, ^c^, ^d^	12.731	<0.001
Self-reported QuENA (score)	6.05 (4.08)	7.20 (4.03)	8.00 (3.07)	5.26 (3.08)	5.70 (2.61)	5.80 (4.47)	0.905	0.479
Informant-reported QuENA (score)	3.03 (2.94)	3.69 (3.12)	4.29 (3.90)	4.94 (4.37)	7.12 (5.19)^a^	7.56 (4.28)	3.295	0.008

Values are mean (SD). *F* and *p* values refer to the main effect across all groups. The analysis was controlled for age, gender, education, and life-space mobility.

Key: CN, cognitively normal; SCD, subjective cognitive decline; aMCI, amnestic mild cognitive impairment; GDS-15, Geriatric Depression Scale 15-item version; BAI, Beck Anxiety Inventory; SSNCQ, Subjective Spatial Navigation Complaints Questionnaire; SBSOD, Santa Barbara Sense of Direction Scale; QuENA, Questionnaire on Everyday Navigational Ability.

^a-d^ Significant differences between the groups based on post-hoc analyses.

a Compared to the CN group.

b Compared to the SCD amyloid negative group.

c Compared to the SCD amyloid positive group.

d Compared to the aMCI amyloid negative group.

**Table 7:** ROC analysis of reported spatial navigation abilities in the memory clinic cohort with biomarkers stratified by amyloid-β status

	Self-reported SSNCQ			Informant-reported SSNCQ			Self-reported SBSOD			Informant-reported SBSOD			Self-reported QuENA			Informant-reported QuENA		
	AUC	p	95% Cl	AUC	p	95% Cl	AUC	p	95% Cl	AUC	p	95% Cl	AUC	p	95% Cl	AUC	p	95% Cl
CN vs. SCD amyloid negative	0.604	0.120	0.475–0.732	0.458	0.584	0.311–0.605	0.574	0.272	0.444–0.703	0.670	0.028	0.525–0.815	0.593	0.164	0.464–0.722	0.564	0.405	0.414–0.714
CN vs. SCD amyloid positive	0.730	0.032	0.551–0.909	0.500	1.000	0.270–0.730	0.550	0.655	0.366–0.735	0.551	0.673	0.312–0.790	0.681	0.108	0.502–0.861	0.594	0.438	0.352–0.836
CN vs. aMCI amyloid negative	0.576	0.256	0.446–0.706	0.536	0.664	0.371–0.701	0.557	0.393	0.424–0.690	0.635	0.110	0.459–0.811	0.458	0.528	0.328–0.588	0.629	0.126	0.463–0.796
CN vs. aMCI amyloid positive	0.656	0.014	0.538–0.774	0.730	0.001	0.609–0.851	0.692	0.002	0.579–0.805	0.872	<0.001	0.783–0.961	0.494	0.925	0.368–0.620	0.776	<0.001	0.666–0.887
CN vs. mild dementia amyloid positive	0.596	0.318	0.406–0.785	0.743	0.026	0.538–0.948	0.596	0.349	0.426–0.767	0.963	<0.001	0.912–1.000	0.480	0.849	0.260–0.701	0.808	0.005	0.621–0.995
SCD amyloid negative vs. SCD amyloid positive	0.640	0.200	0.443–0.836	0.540	0.750	0.290–0.790	0.455	0.696	0.265–0.646	0.387	0.369	0.158–0.616	0.575	0.512	0.363–0.787	0.521	0.869	0.264–0.777
SCD amyloid negative vs. aMCI amyloid negative	0.480	0.774	0.343–0.617	0.576	0.394	0.400–0.751	0.488	0.865	0.350–0.627	0.522	0.809	0.331–0.713	0.358	0.041	0.228–0.488	0.579	0.388	0.395–0.762
SCD amyloid negative vs. aMCI amyloid positive	0.562	0.348	0.434–0.690	0.770	<0.001	0.648–0.891	0.642	0.032	0.519–0.765	0.782	<0.001	0.664–0.900	0.381	0.071	0.252–0.509	0.729	0.003	0.598–0.861
SCD amyloid negative vs. mild dementia amyloid positive	0.496	0.971	0.296–0.697	0.778	0.015	0.569–0.987	0.504	0.967	0.323–0.686	0.854	0.002	0.719–0.990	0.407	0.375	0.185–0.629	0.407	0.375	0.185–0.629
SCD amyloid positive vs. aMCI amyloid negative	0.368	0.227	0.182–0.554	0.368	0.227	0.182–0.554	0.527	0.815	0.358–0.696	0.607	0.414	0.381–0.833	0.607	0.414	0.381–0.833	0.524	0.856	0.270–0.777
SCD amyloid positive vs. aMCI amyloid positive	0.426	0.491	0.236–0.616	0.753	0.036	0.595–0.911	0.705	0.068	0.554–0.856	0.853	0.004	0.729–0.977	0.308	0.087	0.107–0.509	0.685	0.127	0.464–0.906
SCD amyloid positive vs. mild dementia amyloid positive	0.375	0.337	0.125–0.625	0.770	0.072	0.532–1.000	0.513	0.929	0.234–0.791	0.937	0.004	0.819–1.000	0.350	0.286	0.090–0.610	0.690	0.204	0.411–0.970
aMCI amyloid negative vs. aMCI amyloid positive	0.577	0.244	0.447–0.707	0.696	0.018	0.553–0.840	0.629	0.057	0.499–0.752	0.696	0.021	0.540–0.852	0.541	0.537	0.410–0.672	0.634	0.115	0.471–0.797
aMCI amyloid negative vs. mild dementia amyloid positive	0.524	0.807	0.330–0.718	0.716	0.069	0.497–0.936	0.513	0.902	0.339–0.687	0.747	0.040	0.564–0.929	0.534	0.743	0.307–0.761	0.685	0.123	0.465–0.906
aMCI amyloid positive vs. mild dementia amyloid positive	0.440	0.528	0.248–0.632	0.524	0.827	0.309–0.739	0.342	0.122	0.179–0.505	0.520	0.858	0.321–0.718	0.495	0.964	0.255–0.736	0.549	0.654	0.337–0.761

**Key**: CN, cognitively normal; SCD, subjective cognitive decline; aMCI, amnestic mild cognitive impairment; AUC, area under the curve; 95% CI, 95% confidence interval; SSNCQ, Subjective Spatial Navigation Complaints Questionnaire; SBSOD, Santa Barbara Sense of Direction Scale; Questionnaire on Everyday Navigational Ability, QuENA.

### Associations between reported navigation abilities and spatial navigation performance

**Figure 4:**
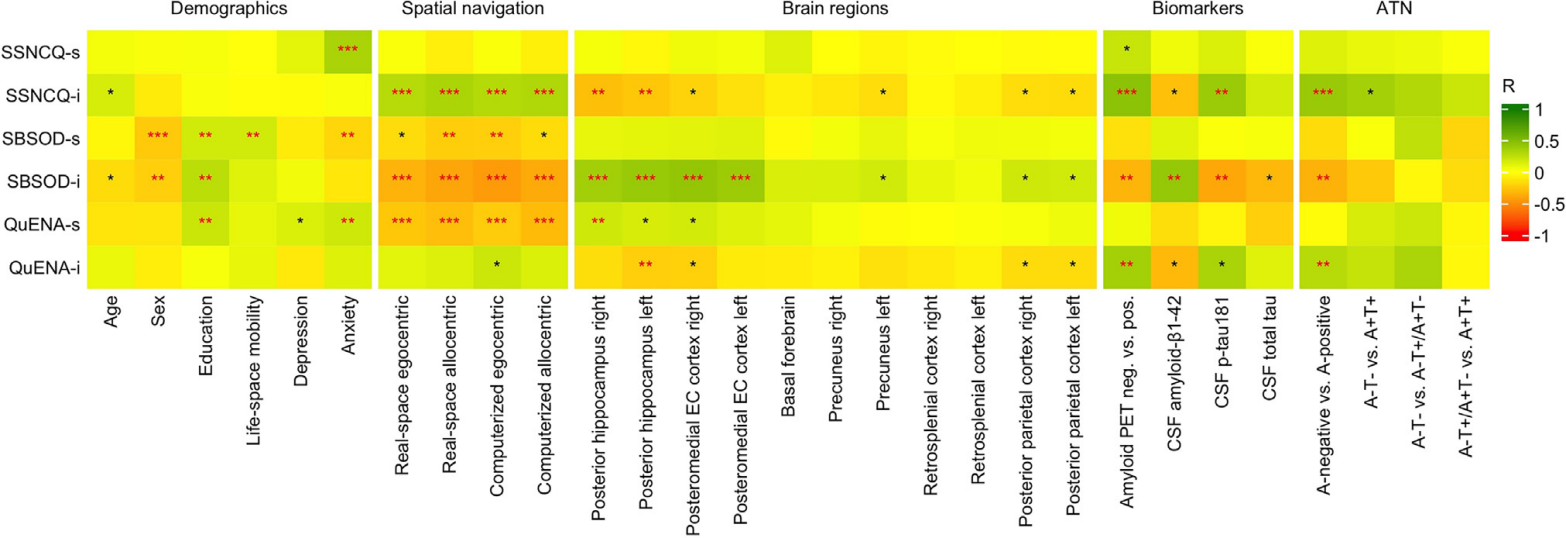
Correlations between reported spatial navigation abilities, demographics, spatial navigation performance, regional brain measures, AD biomarkers, and biomarker status Notes: ∗*p* < 0.05; ∗∗*p* < 0.01; and ∗∗∗*p* < 0.001. Red asterisks indicate significant correlations after adjustment for multiple comparisons using false discovery rate (FDR) correction. Key: SSNCQ, Subjective Spatial Navigation Complaints Questionnaire; SBSOD, Santa Barbara Sense of Direction Scale; QuENA, Questionnaire on Everyday Navigational Ability; -s, self-reported version of the questionnaire; -i, informant-reported version of the questionnaire; ATN, ATN biomarker status; EC, entorhinal cortex; CSF, cerebrospinal fluid; A-negative, amyloid-β negative; A-positive, amyloid-β positive; A, amyloid-β; T, tau; -, normal; +, abnormal.

Correlations between reported navigation abilities and spatial navigation performance in the memory clinic cohort are shown in Figure 4. Informant-reported navigation abilities correlated with navigation performance, in particular, less accurate performance on all navigation tasks was associated with reported poorer navigation abilities as measured by the SSNCQ-i and SBSOD-i, but not the QuENA-i. Self-reported navigation abilities, as measured by the SBSOD-s, and QuENA-s, but not the SSNCQ-s, correlated with navigation performance. Less accurate performance on computerized egocentric and real-space alllocentric tasks was associated with reported poorer navigation abilities as measured by the SBSOD-s. The inverse association with all navigation tasks was found for the QuENA-s, indicating that less accurate spatial navigation performance was associated with reported better spatial navigation abilities.

As shown in Table 8, the associations of informant-reported navigation abilities as measured by the SSNCQ-i and SBSOD-i with navigation performance remained essentially unchanged in the mixed model analyses adjusted for demographics and life-space mobility. The magnitude of the associations between self-reported navigation abilities, as measured by the SBSOD-s, and navigation performance was reduced, with the association with the computerized egocentric task being nonsignificant in the mixed model analyses. For significant interactions of the SSNCQ-i, SBSOD-s, and SBSOD-i scores with group status, the aMCI group accounted for most of the associations between reported navigation abilities and navigation performance, as shown in Table 9.

**Table 8:** Associations between reported spatial navigation abilities and spatial navigation performance in the memory clinic cohort

	Real-space egocentric task			Real-space allocentric task			Computerized egocentric task			Computerized allocentric task		
	β	p	95% CI	β	p	95% CI	β	p	95% CI	β	p	95% CI
**Self-reported SSNCQ**												

Questionnaire score	−0.015	0.749	−0.109–0.078	−0.091	0.054	−0.184–0.001	0.007	0.865	−0.070–00.083	−00.086	0.034	−0.166–−0.006
Questionnaire score∗group interaction	0.061	0.184	−0.029–0.151	0.010	0.832	−0.079–0.098	0.076	0.051	−0.0005–0.152	0.012	0.770	−0.067–0.090

**Informant-reported SSNCQ**												

Questionnaire score	0.201	**<0.001**	0.085–0.318	0.218	**<0.001**	0.107–0.330	0.170	**<0.001**	0.079–0.262	00.191	**<0.001**	0.093–0.289
Questionnaire score∗group interaction	0.135	0.009	0.034–0.235	0.114	0.020	0.018–0.210	0.046	0.278	−0.038–0.130	0.086	0.053	−0.001–0.174

**Self-reported SBSOD**												

Questionnaire score	−0.088	0.087	−0.188–0.013	−0.118	**0.023**	−0.219–−0.017	−0.080	0.054	−10.609–0.002	−0.053	0.232	−0.140–0.034
Questionnaire score∗group interaction	−0.047	0.279	−0.137–0.040	−0.065	0.143	−0.153–0.022	−0.095	0.013	−0.169–−0.020	−0.025	0.524	−0.104–0.053

**Informant-reported SBSOD**												

Questionnaire score	−0.201	**0.001**	−0.322–−0.080	−0.254	**<0.001**	−0.368–−0.140	−0.183	**<0.001**	−0.279–−0.088	−0.185	**<0.001**	−0.286–−0.083
Questionnaire score∗group interaction	−0.209	0.001	−0.334–−0.084	−0.209	<0.001	−0.328–−0.091	−0.111	0.029	−0.210–−0.011	−0.107	0.042	−0.211–−0.004

**Self-reported QuENA**												

Questionnaire score	−0.128	**0.010**	−0.226–−0.031	−0.168	**<0.001**	−0.265–−0.705	−0.105	**0.011**	−0.186–−0.025	−0.165	**<0.001**	−0.249–−0.080
Questionnaire score∗group interaction	−0.057	0.175	−0.139–0.026	−0.035	0.407	−0.117–0.047	−0.027	0.460	−0.098–0.045	−0.041	0.275	−0.114–0.033

**Informant-reported QuENA**												

Questionnaire score	0.089	0.153	−0.033–0.211	0.108	0.078	−0.012–00.229	0.122	**0.015**	0.024–0.219	0.103	0.052	−0.001–0.207
Questionnaire score∗group interaction	0.090	0.096	−0.016–0.197	0.112	0.032	0.010–0.214	0.057	0.199	−0.030–0.145	0.107	0.020	0.017–0.198

The analysis was controlled for age, gender, education, and life-space mobility. *p* values in bold were significant after false discovery rate (FDR) correction. Interactions were not FDR corrected.

**Key:** SCD, subjective cognitive decline; aMCI, amnestic mild cognitive impairment; β, regression coefficient; 95% CI, 95% confidence interval; GDS-15, Geriatric Depression Scale 15-item version; BAI, Beck Anxiety Inventory; SSNCQ, Subjective Spatial Navigation Complaints Questionnaire; SBSOD, Santa Barbara Sense of Direction Scale; QuENA, Questionnaire on Everyday Navigational Ability.

**Table 9:** Associations between reported spatial navigation abilities and spatial navigation performance stratified by groups in the memory clinic cohort

	Real-space egocentric task			Real-space allocentric task			Computerized egocentric task			Computerized allocentric task		
	β	p	95% CI	β	p	95% CI	β	p	95% CI	β	p	95% CI
**Self-reported SSNCQ**												

SCD	N.A.	N.A.	N.A.	N.A.	N.A.	N.A.	N.A.	N.A.	N.A.	N.A.	N.A.	N.A.
aMCI	N.A.	N.A.	N.A.	N.A.	N.A.	N.A.	N.A.	N.A.	N.A.	N.A.	N.A.	N.A.
Mild dementia	N.A.	N.A.	N.A.	N.A.	N.A.	N.A.	N.A.	N.A.	N.A.	N.A.	N.A.	N.A.

**Informant-reported SSNCQ**												

SCD	−0.110	0.284	−0.313–0.094	0.001	0.995	−0.168–0.169	N.A.	N.A.	N.A.	N.A.	N.A.	N.A.
aMCI	0.160	0.037	0.010–0.304	0.135	0.071	−0.012–0.282	N.A.	N.A.	N.A.	N.A.	N.A.	N.A.
Mild dementia	0.004	0.593	−0.470–0.281	0.117	0.409	−0.188–0.422	N.A.	N.A.	N.A.	N.A.	N.A.	N.A.

**Self-reported SBSOD**												

SCD	N.A.	N.A.	N.A.	N.A.	N.A.	N.A.	0.098	0.097	−0.018–0.215	N.A.	N.A.	N.A.
aMCI	N.A.	N.A.	N.A.	N.A.	N.A.	N.A.	−0.150	0.003	−0.246–−0.053	N.A.	N.A.	N.A.
Mild dementia	N.A.	N.A.	N.A.	N.A.	N.A.	N.A.	−0.281	0.164	−0.678–0.116	N.A.	N.A.	N.A.

**Informant-reported SBSOD**												

SCD	0.094	0.349	−0.106–0.294	−0.054	0.514	−0.221–0.112	0.099	0.024	−0.056–0.254	−0.026	0.783	−0.214–0.162
aMCI	−0.163	0.032	−0.311–−0.014	−0.204	0.005	−0.344–−0.064	−0.164	0.004	−0.273–−0.056	−0.122	0.040	−0.238–−0.006
Mild dementia	0.131	0.655	−0.501–0.763	−0.128	0.578	−0.622–0.367	0.223	0.145	−0.077–524	−0.038	0.752	−0.292–0.217

**Self-reported QuENA**												

SCD	N.A.	N.A.	N.A.	N.A.	N.A.	N.A.	N.A.	N.A.	N.A.	N.A.	N.A.	N.A.
aMCI	N.A.	N.A.	N.A.	N.A.	N.A.	N.A.	N.A.	N.A.	N.A.	N.A.	N.A.	N.A.
Mild dementia	N.A.	N.A.	N.A.	N.A.	N.A.	N.A.	N.A.	N.A.	N.A.	N.A.	N.A.	N.A.

**Informant-reported QuENA**												

SCD	N.A.	N.A.	N.A.	−0.001	0.992	−0.130–0.129	N.A.	N.A.	N.A.	−0.007	0.915	−0.147–0.132
aMCI	N.A.	N.A.	N.A.	0.100	0.228	−0.064–0.265	N.A.	N.A.	N.A.	0.042	0.532	−0.091–0.174
Mild dementia	N.A.	N.A.	N.A.	0.071	0.755	−0.422–0.564	N.A.	N.A.	N.A.	0.088	0.359	−0.114–0.290

Associations were analyzed separately for each group if there was a significant interaction between questionnaire score and group status (Table 7).

**Key**: SCD, subjective cognitive decline; aMCI, amnestic mild cognitive impairment; β, regression coefficient; 95% CI, 95% confidence interval; N.A., not applicable; SSNCQ, Subjective Spatial Navigation Complaints Questionnaire; SBSOD, Santa Barbara Sense of Direction Scale; QuENA, Questionnaire on Everyday Navigational Ability.

### Associations of reported navigation abilities with demographics, volume/thickness of AD-related brain regions, AD biomarkers, and biomarker status

Correlations of reported navigation abilities with demographics, volume/thickness of AD-related brain regions, AD biomarkers, and biomarker status in the memory clinic cohort are shown in Figure 4. Self-reported and informant-reported navigation abilities, as measured by the SBSOD-s and SBSOD-i correlated with gender and education, with the SBSOD-i additionally correlated with life-space mobility. Specifically, female gender, lower education, and more limited life-space mobility were associated with reported poorer navigation abilities. Self-reported, but not informant-reported, navigation abilities correlated with anxiety symptoms. Informant-reported navigation abilities correlated with volumes of AD-related brain regions, but self-reported navigation abilities did not (except for a single inverse correlation). As shown in Figure 4, smaller posterior hippocampal volume was associated with reported poorer navigation abilities, as measured by the SSNCQ-i and SBSOD-i (both hemispheres) or the QuENA-i (left hemisphere only). In addition, smaller volume of the posteromedial entorhinal cortex was associated with reported poorer navigation abilities, as measured by the SBSOD-i. As shown in Table 10, these associations remained significant in regression analyses adjusted for demographics and life-space mobility, except for the association between left posterior hippocampal volume and the SSNCQ-i score. In addition, the association between volume of the right posteromedial entorhinal cortex and the QuENA-i score was found to be significant in these analyses. Most of the associations between regional brain atrophy and informant-reported poorer navigation abilities were mediated by less accurate spatial navigation performance, as shown in Table 11.

**Table 10:** Associations between reported spatial navigation abilities and regional brain measures in the memory clinic cohort

	Self-reported SSNCQ			Informant-reported SSNCQ			Self-reported SBSOD			Informant-reported SBSOD			Self-reported QuENA			Informant-reported QuENA		
	β	p	95% Cl	β	p	95% Cl	β	p	95% Cl	β	p	95% Cl	β	p	95% Cl	β	p	95% Cl
Posterior hippocampus right	0.058	0.495	−0.108–0.224	−0.323	**<0.001**	−0.511–−0.134	0.001	0.986	−0.154–0.157	0.301	**0.001**	0.120–0.483	0.107	0.178	−0.049–0.264	−0.204	0.035	−0.393–−0.015
Posterior hippocampus left	−0.046	0.587	−0.213–0.121	−0.265	0.008	−0.458–−0.072	0.029	0.716	−0.127–0.185	0.357	**<0.001**	0.178–0.536	0.073	0.366	−0.086–0.231	−0.271	**0.005**	−0.458–−0.083
Posteromedial entorhinal cortex right	0.082	0.311	−0.078–0.242	−0.251	0.008	−0.436–−0.066	−0.014	0.851	−0.164–0.135	0.368	**<0.001**	0.198–0.538	0.100	0.193	−0.051–0.251	−0.290	**0.002**	−0.468–−0.111
Posteromedial entorhinal cortex left	0.069	0.390	−0.089–0.227	−0.176	0.062	−0.361–0.009	0.002	0.983	−0.146–0.149	0.312	**<0.001**	0.141–0.483	0.054	0.478	−0.096–0.203	−0.149	0.106	−0.330–0.032
Basal forebrain	0.217	0.022	0.032–0.403	0.002	0.984	−0.221–0.225	−0.096	0.280	−0.271–0.079	0.062	0.565	−0.151–0.276	0.027	0.763	−0.151–0.205	−0.074	0.504	−0.291–0.144
Precuneus right	−0.012	0.876	−0.165–0.141	−0.066	0.470	−0.247–0.114	0.036	0.620	−0.107–0.179	0.115	0.187	−0.057–0.287	−0.002	0.974	−0.147–0.142	−0.056	0.529	−0.232–0.120
Precuneus left	−0.057	0.466	−0.209–0.096	−0.115	0.206	−0.295–0.064	0.108	0.136	−0.034–0.249	−0.174	0.046	0.003–0.344	−0.071	0.331	−0.216–0.073	−0.143	0.107	−0.318–0.031
Retrosplenial cortex right	0.046	0.546	−0.104–0.196	−0.123	0.170	−0.301–0.054	0.033	0.644	−0.107–0.173	0.165	0.055	−0.004–0.333	0.135	0.061	−0.007–0.276	−0.140	0.110	−0.313–0.032
Retrosplenial cortex left	0.069	0.389	−0.088–0.225	−0.243	0.009	−0.425–−0.062	0.057	0.440	−0.089–0.204	0.160	0.076	−0.017–0.336	0.112	0.136	−0.036–0.260	−0.154	0.092	−0.334–0.025
Posterior parietal cortex right	−0.031	0.702	−0.189–0.128	−0.112	0.236	−0.299–0.074	0.063	0.402	−0.085–0.211	0.187	0.038	0.011–0.364	−0.009	0.909	−0.159–0.141	−0.177	0.055	−0.357–0.004
Posterior parietal cortex left	−0.018	0.820	−0.176–0.139	−0.109	0.244	−0.294–0.076	0.057	0.442	−0.089–0.204	0.175	0.051	−0.001–0.350	0.028	0.711	−0.121–0.177	−0.171	0.061	−0.350–0.008

*p* values in bold were significant after false discovery rate (FDR) correction.

**Key**: β, regression coefficient; 95% CI, 95% confidence interval; SSNCQ, Subjective Spatial Navigation Complaints Questionnaire; SBSOD, Santa Barbara Sense of Direction Scale; QuENA, Questionnaire on Everyday Navigational Ability.

**Table 11:** Path analysis of the associations between regional brain measures, spatial navigation performance and reported spatial navigation abilities in the memory clinic cohort

	Total effect (c)	Direct effect (c')	Indirect effect (a∗b)	
	β	β	β	95% CI
Posterior hippocampus right → Real-space allocentric → SSNCQ-i	−0.417∗∗∗	−0.320∗∗	−0.097	−0.120–−0.024
Posterior hippocampus right → Computerized allocentric → SSNCQ-i	−0.437∗∗∗	−0.341∗∗	−0.097	−0.206–−0.013
Posterior hippocampus right → Real-space allocentric → SBSOD-i	0.320∗∗	0.211∗	0.109	0.032–0.213
Posterior hippocampus left → Real-space allocentric → SBSOD-i	0.371∗∗∗	0.246∗	0.125	0.034–0.248
Posteromedial entorhinal cortex right → Real-space allocentric → SBSOD-i	0.298∗∗∗	0.240∗∗	0.057	0.009–0.128
Posteromedial entorhinal cortex left → Real-space allocentric → SBSOD-i	0.0261∗∗	0.212∗	0.049	−0.010–0.128

**Figure 5:**
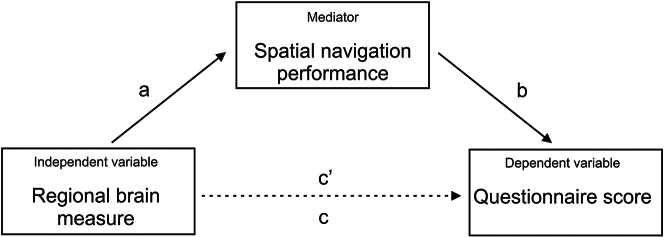
Path analysis In the path analysis, regional brain measure is the independent variable, spatial navigation questionnaire score is the dependent variable and spatial navigation performance is the mediator. The “c” indicates the total effect of the independent variable on the dependent variable, the “c’” indicates the direct effect of the independent variable on the dependent variable controlled for the mediator, and “a∗b” indicate the indirect effect of the independent variable on the dependent variable through the mediator.

∗*p* < 0.05; ∗∗*p* < 0.01; and ∗∗∗*p* < 0.001. The path analysis model is shown in Figure 5.

**Key**: β, regression coefficient; 95% CI, 95% confidence interval; SSNCQ, Subjective Spatial Navigation Complaints Questionnaire; SBSOD, Santa Barbara Sense of Direction Scale; QuENA, Questionnaire on Everyday Navigational Ability.

Informant-reported, but not self-reported, navigation abilities correlated with greater AD biomarker abnormalities and A-positive biomarker status, as shown in Figure 4. Amyloid PET positivity was associated with reported poorer navigation abilities, as measured by the SSNCQ-i, SBSOD-i, and QuENA-i. In addition, higher CSF p-tau_181_ levels were associated with reported poorer navigation abilities, as measured by the SSNCQ-i and SBSOD-i, and lower CSF amyloid-β_1-42_ levels were associated with reported poorer navigation abilities, as measured by the SBSOD-i. No significant associations were observed between CSF total tau levels and informant-reported navigation abilities. A-positive status, but not the A+T+ status, was associated with reported poorer navigation abilities, as measured by the SSNCQ-i, SBSOD-i, and QuENA-i. As shown in Tables 12 and 13, these associations remained significant in regression analyses adjusted for demographics and life-space mobility, except for the association between A-positive status and the QuENA-i score.

**Table 12:** Associations between reported spatial navigation abilities and AD biomarkers in the memory clinic cohort

	Self-reported SSNCQ			Informant-reported SSNCQ			Self-reported SBSOD			Informant-reported SBSOD			Self-reported QuENA			Informant-reported QuENA		
	β	p	95% Cl	β	p	95% Cl	β	p	95% Cl	β	p	95% Cl	β	p	95% Cl	β	p	95% Cl
Amyloid PET negative vs. positive	0.454	0.042	0.016–0.892	0.921	**<0.001**	0.435–1.407	−0.402	0.054	−0.812–0.007	−0.738	**0.003**	−1.221–−0.255	0.161	0.451	−0.262–0.584	0.699	**0.006**	0.204–1.194
CSF amyloid-β_1-42_	0.058	0.669	−0.211–0.326	−0.277	0.073	−0.581–0.027	0.131	0.300	−0.120–0.381	0.441	**0.002**	0.170–0.713	−0.156	0.221	−0.410–0.097	−0.302	0.045	−0.597–−0.007
CSF p-tau_181_	0.132	0.339	−0.142–0.407	0.397	**0.011**	0.096–0.697	0.029	0.825	−0.231–0.289	−0.404	**0.007**	−0.691–−0.117	−0.047	0.723	−0.311–0.217	0.336	0.030	0.035–0.637
CSF total tau	0.005	0.971	−0.287–0.297	0.153	0.367	−0.187–0.493	0.055	0.691	−0.220–0.329	−0.330	0.040	−0.644–−0.016	−0.217	0.115	−0.489–0.055	0.119	0.475	−0.216–0.455

*p* values in bold were significant after false discovery rate correction.

**Key:** β, regression coefficient; 95% CI, 95% confidence interval; SSNCQ, Subjective Spatial Navigation Complaints Questionnaire; SBSOD, Santa Barbara Sense of Direction Scale; QuENA, Questionnaire on Everyday Navigational Ability; CSF, cerebrospinal fluid; p-tau_181_, phosphorylated tau_181_.

**Table 13:** Associations between reported spatial navigation abilities and biomarker status in the memory clinic cohort

	Self-reported SSNCQ			Informant-reported SSNCQ			Self-reported SBSOD			Informant-reported SBSOD			Self-reported QuENA			Informant-reported QuENA		
	β	p	95% Cl	β	p	95% Cl	β	p	95% Cl	β	p	95% Cl	β	p	95% Cl	β	p	95% Cl
A-negative v.s. A-positive	0.245	0.184	−0.118–0.609	**0.770**	**<0.001**	**0.365–1.174**	−0.390	0.023	−0.725–−0.054	**−0.720**	**<0.001**	**−1.115–-0.326**	−0.022	0.899	−0369–0.325	0.518	0.015	0.103–0.933
A-T- v.s. A + T+	0.096	0.575	−0.247–0.440	0.385	0.040	0.018–0.752	−0.024	0.883	−0.350–0.302	−0.331	0.075	−0.698–0.035	0.150	0.360	−0.177–0.478	0.232	0.225	−0.150–0.614
A-T- v.s. A-T+/A + T-	0.167	0.687	−0.676–1.010	0.617	0.302	−0.628–1.863	0.406	0.285	−0.358–1.170	−0.262	0.667	−1.564–1.041	0.335	0.386	−0.446–1.116	0.682	0.256	−0.571–1.936
A-T+/A + T- v.s. A + T+	0.029	0.929	−0.619–0.677	0.439	0.234	−0.297–1.176	−0.402	0.184	−1.003–0.198	−0.325	0.365	−1.046–0.395	−0.073	0.815	−0.694–0.549	−0.117	0.749	−0.856–0.622

*p* values in bold were significant after false discovery rate (FDR) correction.

**Key**: β, regression coefficient; 95% CI, 95% confidence interval; SSNCQ, Subjective Spatial Navigation Complaints Questionnaire; SBSOD, Santa Barbara Sense of Direction Scale; QuENA, Questionnaire on Everyday Navigational Ability; A, amyloid; T, tau.

## Discussion

The present study is the first to compare self- and informant-reported spatial navigation abilities and their associations with several objective measures of spatial navigation, brain atrophy, and biomarkers across the AD continuum. Results showed that informant-reported, but not self-reported, spatial navigation questionnaires discriminated participants with aMCI and mild dementia from CN participants, and also discriminated amyloid-β positive aMCI participants from amyloid-β negative aMCI and CN participants, whereas the latter groups were similar to each other. In participants from the memory clinic cohort, informant-reported poorer spatial navigation abilities were associated with less accurate performance on real-space and computerized egocentric and allocentric navigation tasks, whereas self-reported navigation abilities were not (except for a single weak association). Next, greater atrophy of AD-related brain regions, including the posterior hippocampus and posteromedial entorhinal cortex, greater AD biomarker abnormalities, and A-positive status were associated with informant-reported, but not self-reported, poorer spatial navigation abilities. These results suggested that informant-reported information about spatial navigation abilities is more reliable for diagnostic classification, and is more strongly associated with spatial navigation performance, AD-related structural brain changes, and AD biomarkers.

### Spatial navigation questionnaires as a tool to differentiate between clinically and biomarker-defined participants

We have previously shown that individuals with SCD, MCI, and dementia report spatial navigation complaints on the SSNCQ-s more frequently than CN older adults.^33^ Other studies have shown that the QuENA can identify individuals with AD dementia who experience episodes of getting lost^34^ and that the QuENA-i in particular can predict the occurrence of these episodes over a three-year period.^35^ The present study was the first to comprehensively evaluate the potential of several self- and informant-reported spatial navigation questionnaires (i.e., the SSNCQ, SBSOD, and QuENA) to discriminate between CN older adults and participants from the memory clinic cohort, including individuals with SCD, aMCI, and mild dementia defined by clinical criteria and stratified by biomarkers. Results showed that informant-reported questionnaires discriminated CN participants from those with aMCI and mild dementia (all questionnaires), SCD from mild dementia (all questionnaires), SCD from aMCI (the SSNCQ-i and SBSOD-i), and CN participants from those with SCD (the SBSOD-i). The ability of the informant-reported questionnaires to discriminate CN older adults from those with aMCI or mild dementia, as measured by AUC, was satisfactory to good (range 0.65–0.75) and good to excellent (range 0.77–0.91), respectively. In contrast, none of the self-reported spatial navigation questionnaires discriminated between clinically defined participants. This is consistent with our previous findings that SCD and MCI individuals had similar self-reported spatial navigation abilities on the SSNCQ-s,^36^ and with studies showing that informant-reported, as opposed to self-reported, memory or cognitive decline had a greater ability to discriminate between CN older adults and individuals with cognitive impairment,^44^^,^^45^ and to predict progression to MCI and dementia.^41^^,^^42^^,^^43^^,^^47^ Thus, the higher discriminatory and predictive ability of informant-reported than self-reported questionnaires suggested that insight into the presence of cognitive deficits may not be maintained even in the very early stages of AD. In fact, the previous study showed that aMCI individuals may have impaired insight into their cognitive deficits to the same extent as those with mild dementia.^50^ Results also showed that the informant-reported questionnaires SSNCQ-i and SBSOD-i discriminated amyloid-β positive aMCI participants from amyloid-β negative aMCI, amyloid-β negative SCD, and CN participants, who were similar to each other. The ability of the SSNCQ-i and SBSOD-i to discriminate CN older adults and participants with amyloid-β negative aMCI from those with amyloid-β positive aMCI, as measured by AUC, was good to very good (range 0.73–0.87) and good (i.e., 0.70), respectively. The QuENA-i discriminated only between CN and amyloid-β positive aMCI participants with a good AUC of 0.78. Thus, the discriminative ability of informant-reported spatial navigation questionnaires was only slightly lower than previously reported for objective measures of spatial navigation.^51^ In contrast, the self-reported spatial navigation questionnaires did not discriminate between biomarker-defined participants, except for the SBSOD-s, which discriminated CN from amyloid-β positive aMCI participants with a satisfactory AUC of 0.69. This is broadly consistent with previous research showing that MCI individuals with positive AD biomarkers underestimate their cognitive deficits, while those with negative AD biomarkers overestimate their cognitive difficulties relative to their informants.^52^ The tendency to underreport cognitive difficulties in MCI individuals with AD may be due to decreased awareness of cognitive dysfunction (i.e., anosognosia) or an inability to accurately assess one’s own cognitive abilities.^53^ A previous study suggested that self-reported spatial navigation abilities may discriminate CN older adults from those with preclinical AD.^28^ Due to the very small number of amyloid-β positive SCD participants, we were not able to show whether they could be distinguished from amyloid-β negative SCD or CN participants using self- and informant-reported questionnaires, and future research is needed to address this issue. Overall, the results demonstrated the promising role of informant-reported spatial navigation abilities in screening clinically and biomarker-defined cognitively impaired individuals recruited from the memory clinic and the very limited screening potential of self-reported spatial navigation abilities. These findings are broadly consistent with previous research showing that even individuals with MCI and mild dementia, especially those with positive AD biomarkers, tend to overestimate their cognitive abilities due to lack of insight, whereas SCD individuals may underestimate their cognitive abilities relative to their informants due to mood and personality traits.^52^^,^^54^^,^^55^ Taken together, these findings highlight the important role of informants as a critical source of information about cognitive abilities, including spatial navigation, in memory clinic cohorts.

### Reported navigation abilities and their associations with spatial navigation performance

Previous studies have shown that spatial navigation questionnaires are reliable predictors of spatial navigation performance in young, middle-aged, and older adults.^29^^,^^30^^,^^31^^,^^56^ To extend current knowledge, the present study examined the potential of self- and informant-reported spatial navigation abilities to reflect objectively measured egocentric and allocentric navigation performance in individuals from the memory clinic cohort. For this purpose, we used the hMWM, which has been designed and successfully used to assess egocentric and allocentric navigation strategies,^57^^,^^58^ has shown associations between navigation and the brain structures implicated in the spatial processing and impairment in early AD,^12^^,^^59^ and provides a high degree of control over the experimental situation. Results showed that performance on the real-space and computerized versions of egocentric and allocentric navigation tasks was predicted by the scores on informant-reported questionnaires, specifically the SSNCQ-i and SBSOD-i, but not the QuENA-i. These associations were mostly driven by individuals with aMCI. In contrast, there was a single weak association between self-reported navigation abilities, as measured by the SBSOD-s, and real-space allocentric navigation performance, whereas the associations of the SSNCQ-s and QuENA-s scores with spatial navigation performance, were nonsignificant or reversed, respectively, similar to the previous study in real-world settings with prodromal AD and mild AD dementia participants.^37^ These findings add to previous research that found no associations between self-reported spatial navigation abilities and objective measures of spatial navigation in CN individuals with positive and negative AD biomarkers in CSF,^28^ and studies showing that the associations with objective measures of memory are stronger for informant-reported than for self-reported memory or cognitive decline, particularly in individuals with MCI and mild AD dementia.^45^^,^^46^^,^^47^^,^^48^ Overall, these findings suggested that specific informant-reported rather than self-reported questionnaires may be a reliable source of information about cognitive difficulties, including spatial navigation deficits, in cognitively impaired individuals within memory clinic cohorts, which may reflect the fact that as the disease progresses, people become less aware of their cognitive deficits due to increasing levels of anosognosia, leading to unreliable self-reports.^55^ It would be very interesting to compare specific questions in individual questionnaires that should reflect different navigation strategies in the real world with objectively measured navigation strategies in the hMWM. However, these questions focus on large-scale spatial abilities, specifically route learning and map preference for navigation in the SBSOD, and landmark agnosia, egocentric disorientation (which more closely reflects route learning), heading disorientation, and inattention in the QuENA, which are not directly measured by the hMWM. In addition, the hMWM involves navigation in a small, open space and therefore may have limited ecological validity for human navigation. To address this limitation, spatial navigation tasks based on complex realistic indoor^7^^,^^56^^,^^60^ and outdoor^60^^,^^61^^,^^62^^,^^63^^,^^64^^,^^65^ environments have recently been designed and adapted to more comprehensively measure spatial navigation behavior, including route learning, wayfinding, cognitive mapping, perspective taking, path integration, and landmark placement. Future research should compare spatial navigation performance in these complex, realistic environments with spatial navigation questionnaire scores and specific questions to more accurately assess the associations between real-world navigation and reported navigation abilities.

### Reported navigation abilities and their associations with atrophy of AD-related brain regions

This is the first study to examine the relationship between self- and informant-reported navigation abilities and brain atrophy in individuals from the memory clinic cohort. We measured the volume or thickness of six brain regions (i.e., the posterior hippocampus, posteromedial entorhinal cortex, basal forebrain, retrosplenial cortex, precuneus, and posterior parietal cortex) involved in spatial navigation^9^^,^^10^^,^^14^^,^^15^^,^^16^^,^^17^ and associated with early accumulation of AD pathology.^18^^,^^19^ Results showed that greater atrophy of the posterior hippocampus and posteromedial entorhinal cortex was associated with informant-reported, but not self-reported, poorer navigation abilities. The associations were partially explained by objectively measured allocentric navigation deficits. This is broadly consistent with previous studies showing that greater AD-related atrophy of the hippocampus and entorhinal cortex is more strongly associated with informant-reported than self-reported cognitive decline, particularly in individuals with MCI and mild AD dementia.^45^^,^^46^^,^^47^^,^^49^ The results of the current study, as well as the previous studies, thus suggested that informants can perceive objective cognitive changes associated with the neurodegenerative processes underlying AD and that specific informant-reported cognitive questionnaires, including those focused on spatial navigation difficulties, can reliably reflect AD-associated medial temporal lobe atrophy in memory clinic cohorts, but further studies are needed to confirm these findings.

### Reported navigation abilities and their associations with AD biomarkers and biomarker status

Previous studies have suggested that self-reported greater spatial navigation difficulties, as measured by the SBSOD-s, are associated with lower CSF amyloid-β_1-42_ levels in CN older adults.^28^^,^^32^ More recently, these findings have not been replicated using the Everyday Cognition Scale, as self- and informant-reported spatial navigation difficulties were not associated with increased CSF p-tau_181_/amyloid-β_1-42_ ratios in CN older adults.^66^ The present study examined the associations of self-reported and informant-reported spatial navigation abilities with CSF biomarker abnormalities, amyloid PET positivity, and biomarker status across the clinical continuum of participants in the memory clinic cohort, including those with SCD, aMCI, and mild dementia. Results showed that A-positive status and greater AD biomarker abnormalities, including amyloid PET positivity, lower CSF amyloid-β_1-42_, and higher CSF p-tau_181_ levels, were associated with informant-reported, but not self-reported, poorer spatial navigation abilities, particularly as measured by the SSNCQ-i and SBSOD-i. These findings are consistent with a recent study linking informant-reported greater memory decline, corrected for the effect of self-reported memory decline, with increased amyloid-β burden on PET in individuals across the AD clinical continuum,^46^ as well as a previous study showing that greater amyloid-β deposition on PET, lower CSF amyloid-β_1-42_, and higher CSF p-tau_181_ levels are more strongly associated with informant-reported than self-reported cognitively relevant functional abilities in individuals across the AD continuum, particularly those with MCI.^45^ Therefore, informant-reported rather than self-reported greater cognitive difficulties, including those with spatial navigation, may indicate A-positive status and greater AD biomarker abnormalities in memory clinic cohorts.

### Limitations of the study

This study has several limitations. First, informant-reported spatial navigation questionnaires were not available for ⁓29% participants, which may reduce the statistical power of the observed between-group differences and associations with objective spatial navigation measures, brain atrophy and biomarkers. It is worth noting that that there were essentially no differences between clinically and biomarker-defined participants with and without completed informant versions of the questionnaires. Second, some participants and informants left some questionnaire items unanswered and some spatial navigation trials were missing due to technical difficulties. However, missing data (<10%) were imputed using a multiple imputation method that pooled five generated datasets to reduce bias in the data estimate. Third, CSF biomarker assessment and amyloid PET imaging were not performed in CN participants. Therefore, we cannot exclude the possibility that some of them had preclinical AD. However, we used strict inclusion criteria to minimize the risk of recruiting individuals with preclinical AD. Fourth, only a subset of participants from the memory clinic cohort had CSF biomarkers and amyloid PET imaging, which may reduce the statistical power of the observed associations between biomarkers and reported spatial navigation abilities, and differences between the groups stratified by amyloid-β status, particularly the amyloid-β positive SCD and mild dementia groups. Fifth, the participant groups were not matched for demographics, with the control group being younger and having a higher proportion of women, especially compared to the mild dementia group. This may have influenced our results, although all analyses were controlled for demographics. Sixth, the hMWM is a widely used and very useful paradigm for studying egocentric and allocentric navigation strategies, but its main drawback is that it involves navigation in a small, open space and thus its ecological validity is limited. Therefore, future studies using ecologically valid navigation tasks involving navigation in larger spaces with distinct landmarks and visual obstacles (e.g., virtual cities or landscapes) that mimic navigation in real-world environments^61^^,^^67^ are needed to comprehensively assess the relationship between reported navigation abilities and spatial navigation performance. Seventh, the dichotomous visual reading of amyloid PET did not allow quantifying amyloid-β accumulation and examining its association with reported spatial navigation abilities. Eighth, existing atlases define the retrosplenial cortex based on functional activation,^68^ which may not fully correspond to the known anatomical boundaries. This may have influenced the associations observed in the present study. Ninth, the lack of specific biomarkers and the cross-sectional design did not allow identification of underlying disease in participants with negative AD biomarkers, and therefore longitudinal follow-up is needed.

### Conclusions

Using three different spatial navigation questionnaires, the present study demonstrated that informant-reported questionnaires discriminated participants with aMCI and mild dementia from CN older adults, and amyloid-β positive aMCI participants from amyloid-β negative aMCI and CN participants. Next, in the memory clinic cohort, informant-reported poorer navigation abilities were associated with objectively measured worse egocentric and allocentric navigation and greater atrophy of AD-related brain regions (i.e., the posterior hippocampus and posteromedial entorhinal cortex). Finally, informant-reported poorer spatial navigation abilities were associated with greater AD biomarker abnormalities (i.e., lower CSF amyloid-β_1-42_ and higher CSF p-tau_181_ levels, and amyloid PET positivity) and A-positive status in the memory clinic cohort. Therefore, informant-reported spatial navigation questionnaires particularly the SSNCQ-i and SBSOD-i, may be a useful, cost-effective screening tool for early AD in clinical settings that does not require specialized equipment, is easy and quick to administer, and could be used to pre-select at-risk individuals for biomarker assessment.

## STAR★Methods

### Key resources table

**Table:** 

REAGENT or RESOURCE	SOURCE	IDENTIFIER
**Deposited data**		

Raw and analyzed data	This paper	https://osf.io/q25gh/?view_only=2e7a3746b50f473eab5eb9da5e7812f1

**Software and algorithms**		

Codes for the R software	R project	https://osf.io/q25gh/?view_only=2e7a3746b50f473eab5eb9da5e7812f1

### Resource availability

#### Lead contact

Jan Laczó, Memory Clinic, Department of Neurology, Second Faculty of Medicine, Charles University and Motol University Hospital, V Uvalu 84, Praha 5 – Motol, 150 06, Czechia. Tel.: +420 224 436 809; E-mail: jan.laczo@lfmotol.cuni.cz.

#### Materials availability

This study did not generate new unique reagents or other materials.

#### Data and code availability

• Data: The dataset with variables analyzed during the current study is available via the OSF repository: https://osf.io/q25gh/?view_only=2e7a3746b50f473eab5eb9da5e7812f1.
• Codes: Codes for the R software used to plot the results are available at: https://osf.io/q25gh/?view_only=2e7a3746b50f473eab5eb9da5e7812f1.
• Other: Any additional information and dataset required to reanalyze the data reported in this paper is available from the lead contact upon request.

### Experimental model and study participant details

#### Recruitment and inclusion criteria

This study included 262 participants from the CBAS cohort.^69^ 65% of participants were females. All participants were white and of Czech ethnicity. Information on the participants' ancestry was not available. Participants from the memory clinic cohort (n = 221) were recruited in the Memory Clinic of the Charles University, Second Faculty of Medicine, and Motol University Hospital, Prague, Czech Republic. They were referred to the Memory Clinic by general practitioners and neurologists for memory complaints reported by participants themselves, their informants, or health professionals. CN older adults (n = 41) were recruited from the University of the Third Age, senior centers or were relatives of memory clinic participants and hospital staff. All participants underwent clinical evaluation, including routine blood tests, cognitive assessment, brain magnetic resonance imaging (MRI), spatial navigation assessment, and completed questionnaires of spatial navigation abilities. The participants from the memory clinic cohort (i.e., those with SCD, aMCI and dementia) underwent biomarker assessment including measurement of CSF amyloid-β_1-42_, p-tau_181,_ and total tau or amyloid PET imaging or both. The participants signed an informed consent form approved by the Ethics Committee of the Motol University Hospital (study approval number EK701/16).

(i) Participants with SCD (n = 76) met the clinical criteria for SCD^26^ including self-experienced persistent cognitive decline within the past 5 years compared with a previously normal cognitive status, unrelated to an acute event and normal age-, gender and education-adjusted performance on standardized cognitive tests. The participants had normal activities of daily living (i.e., Clinical Dementia Rating [CDR] global score ≤ 0.5).^70^
(ii) Participants with aMCI (n = 117) met the clinical criteria for aMCI^71^ including memory complaints, evidence of memory impairment (i.e., > 1.5 standard deviations [SDs] below the mean of the age-, gender- and education-adjusted norms in any memory test), and generally intact instrumental activities of daily living (i.e., CDR global score ≤ 0.5).
(iii) Participants with mild dementia (n = 28) met the clinical criteria for AD dementia^72^ with evidence of progressive cognitive impairment in at least two cognitive domains including memory (i.e., > 1.5 SDs below the mean of the age-, gender- and education adjusted norms in any memory test and in at least one other non-memory cognitive test) and significant impairment in instrumental activities of daily living (i.e., CDR global score 1).
(iv) CN participants (n = 41) did not report any cognitive complaints, had normal age-, gender- and education-adjusted performance on standardized cognitive tests, no family history of AD or other type of dementia in first-degree relatives, and no evidence of hippocampal atrophy on MRI, as confirmed by visual rating of medial temporal lobe atrophy using the previously established age-specific cut-off scores (i.e., score < 2 in participants < 75 years and score < 3 in participants ≥ 75 years).^73^ These criteria were applied to minimize the risk of including participants at increased risk of AD (i.e., individuals with SCD, hippocampal atrophy or positive family history of AD).

Demographic data for study participants are shown in Table 1. Participants from the memory clinic cohort who underwent biomarker assessment (n = 137) were also categorized into amyloid-β negative and positive groups (Table 2) and eight biomarker profiles (Table 3) according to amyloid-β status and the AT(N) framework,^3^ respectively. We used CSF amyloid-β_1-42_ and amyloid-β phase on amyloid PET for “A”, CSF p-tau_181_ for “T”, and CSF total tau and perfusion phase on amyloid PET for “N”, with each biomarker dichotomized as normal (-) or abnormal (+). Biomarkers were available for 44 SCD, 81 aMCI, and 12 dementia participants. Specifically, CSF biomarkers were analyzed in 10 SCD, 50 aMCI, and 9 dementia participants. Amyloid PET imaging was performed in 38 SCD, 56 aMCI, and 6 dementia participants. Both, CSF biomarkers and amyloid PET imaging, were available for 5 SCD, 25 aMCI, and 2 dementia participants.

#### Exclusion criteria

Participants younger than 55 years, with low visual acuity (< 20/40 [corrected] on visual acuity tests), gait disorders, moderate to severe white matter vascular lesions on MRI (Fazekas score > 2 points), primary brain disorders that may affect cognitive functions, including neurological (i.e., multiple sclerosis, epilepsy, parkinsonian syndromes, frontotemporal lobar degeneration, and a history of traumatic brain injury or stroke) and psychiatric (i.e., psychotic or schizoaffective disorders, major depressive disorder, anxiety disorders, and obsessive compulsive disorder) disorders, systemic diseases that can cause cognitive impairment (i.e., renal, hepatic and cardiac failure, decompensated diabetes, and oncological disease), and a history of alcohol or drug abuse were not included in the study. Participants with dementia (n=4) who did not complete the spatial navigation training task were excluded.

### Method details

#### Spatial navigation assessment

Spatial navigation was assessed using the hMWM, a well-validated method designed for measuring egocentric and allocentric navigation^11^^,^^74^^,^^75^ that has been used in our previous studies.^40^^,^^59^^,^^76^ The hMWM has a computerized and a real-space version. The computerized version represents a map view of the arena projected on a 22-inch computer touch screen and is displayed as a large white circle 290 pixels in diameter with the start position (medium-sized red circle) and two landmarks (red and green lines) on its perimeter, and a small red circle inside the arena representing the goal. The real-space version is performed in a cylindrical arena completely enclosed by a dark blue curtain, 2.9 meters in diameter and 2.8 meters high with eight large numerical displays mounted on the wall at a height of 160 cm from the floor, used to display landmarks and the start position, and eight projectors mounted on the ceiling, used to project the position of the goal on the floor (Figure 1). A television camera with a sampling rate of 25 frames per second, mounted on the ceiling above the center of the arena, was used to capture the position of three infrared light-emitting diodes placed on the top of a hat worn by the participants during the test, and to indicate their location. The participants located an invisible goal in the arena by walking to and standing on the goal and drawing a line from the start position to the goal using a stylus in the real-space and the computerized version, respectively. There were three tasks in the hMWM (Figure 2), allocentric-egocentric (Figure 2A), egocentric (Figure 2B) and allocentric (Figure 2C). The first, the allocentric–egocentric task, was a training task designed to familiarize participants with the testing procedure and involved locating the goal using the start position and two landmarks. The second, the egocentric task, involved locating the goal using the start position without displaying the landmarks. The third, the allocentric task, involved using two landmarks to locate the goal from the starting position unrelated to the goal position. Each task consisted of eight trials and after each trial the correct goal position was displayed to provide feedback. The relative positions (distances and directions) of the goal to the start position or to both landmarks were constant across all trials in all tasks. After each trial, the goal position together with the start position and the positions of two landmarks were rotated in a pseudorandom order and the participants were instructed to go to the new start position on each subsequent trial in all tasks. There was no time limit for locating the goal. Spatial navigation performance, measured as the distance error in centimeters and pixels from the correct goal position in the real-space and the computerized version, respectively, was automatically recorded by the computer. Lower distance error indicated better spatial navigation performance.

#### Self- and informant-reported spatial navigation abilities

Three questionnaires, the Subjective Spatial Navigation Complaints Questionnaire (SSNCQ),^36^ Santa Barbara Sense of Direction Scale (SBSOD)^29^ and Questionnaire on Everyday Navigational Ability (QuENA)^34^ were used to assess both self- and informant-reported spatial navigation abilities. Participants and informants completed the questionnaires prior to the spatial navigation assessment. Informants, who completed the questionnaires, were identified by participants as persons who know them well and interact with them on a regular basis. The most common informants were the spouses and adult children of the participants. Assistance in clarifying questions was provided when needed. Better self-reported and objectively measured cognitive performance is associated with greater life-space mobility,^38^^,^^39^ which may interfere with reported spatial navigation abilities. Therefore, life-space mobility was assessed using the self-reported Life-Space Assessment (LSA) questionnaire^38^ and controlled for in the statistical analyses. The SBSOD, QuENA and LSA were translated from English to Czech by an experienced translator, and then back-translated from Czech to English by a translator blinded to the original English version. The discrepancies were discussed, and the final version was developed based on the consensus between the two translations, to preserve the original meaning as accurately as possible. The SSNCQ, SBSOD, QuENA, and LSA questionnaires are shown in Figures S2–S5 respectively.

##### Subjective Spatial Navigation Complaints Questionnaire

The questionnaire was designed in our memory clinic^36^ to assess the frequency of spatial navigation complaints in everyday life in the past three months. The questionnaire consisted of 15 statements divided into seven section that evaluated: (1) spatial navigation difficulties in different environments, (2) getting-lost events in different environments, (3) decline in spatial navigation abilities in well known places, (4) decline in spatial navigation abilities in relatively less known places, (5) decline in spatial navigation abilities that resulted in seeking more aid, (6) navigation skills in a supermarket as a place challenging for navigation, and (7) impact of navigation difficulties on everyday general functioning. Higher scores indicated worse perception of navigation abilities. The composite score (SSNC composite) was calculated as the sum of all responses. The questionnaire has a high degree of internal consistency (Cronbach’s alpha = 0.89).^36^ The self-reported questionnaire (SSNCQ-s) was unavailable for 4 aMCI participants. The informant-reported questionnaire (SSNCQ-i) was unavailable for 6 CN, 20 SCD, 39 aMCI and 10 mild dementia participants.

##### Santa Barbara Sense of Direction Scale

The SBSOD was designed by Hegarty^29^ as a self-report measure of environmental spatial ability. This scale consisted of 15 statements concerning the ability to navigate in space. The statements were, for example: “I very easily get lost in a new city” and “I am very good at reading maps.” Responses were given on a 1 (strongly agree) to 7 (strongly disagree) scale.^77^ Positively phrased items such as “I am very good at giving directions” were reverse coded (a rating of 1 “strongly agree” was reversed to the score of 7), such that higher scores indicated higher self-reported navigation ability. The composite score (SBSOD composite) was calculated as the average score of all responses. The SBSOD was found to be highly internally consistent (Cronbach’s alpha = 0.88).^29^ The self-reported questionnaire (SBSOD-s) was unavailable for 2 SCD, 5 aMCI and 2 mild dementia participants. The informant-reported questionnaire (SBSOD-i) was unavailable for 6 CN, 20 SCD, 41 aMCI and 9 mild dementia participants.

##### Questionnaire on Everyday Navigational Ability

The QuENA was developed by Pai^34^ to detect spatial navigation impairment in real-world environments. The questionnaire consisted of 10 statements where participants and informants rated on a 4-point scale where a specific symptom would occur (0 = never, 1 = in a less familiar place, 2 = in a moderately familiar place, 3 = in a very familiar place). Higher score indicated a more severe condition. The composite score (QuENA composite) was calculated as the sum of all responses. The questionnaire has a high degree of internal consistency (Cronbach's alpha is 0.87 and 0.91 for the patient and informant versions of the questionnaire, respectively).^34^ The self-reported questionnaire (QuENA-s) was unavailable for 2 SCD, 5 aMCI and 2 mild dementia participants. The informant-reported questionnaire (QuENA-i) was unavailable for 6 CN, 20 SCD, 41 aMCI and 9 mild dementia participants.

##### Life-Space Assessment

The LSA^38^ was used to assess life-space mobility by measuring extent and frequency of movement and also whether any assistance is needed. The questionnaire assessed life-space mobility during the 4 weeks before the administration of the questionnaire. The questionnaire measured mobility in specific life-spaces (i.e., levels: (1) “rooms of your home besides the room where you sleep’’; (2) ‘‘an area outside your home such as your porch, deck or patio, hallway of an apartment building, or garage’’; (3) ‘‘places in your neighborhood, other than your own yard or apartment building’’; (4) ‘‘places outside your neighborhood but within your town’’; and (5) ‘‘places outside your town”). Participants reported the frequency of movement in the specific levels (1 = less than once per week, 2 = 1–3 times per week, 3 = 4–6 times per week, 4 = daily) and the use of assistance from equipment or persons (2 = independent, 1.5 = used equipment, 1 = had personal assistance). Total LSA scores ranged from 0 to 120, with higher scores reflecting greater life-space mobility.^78^

#### Cognitive assessment

Cognitive functions were assessed using the following tests: (1) global cognitive function measured with the Mini-Mental State Examination (MMSE);^79^ (2) verbal memory measured with the Logical Memory (LM) – Immediate and 20-minute Delayed Recall conditions^80^ and the Rey Auditory Verbal Learning Test – trials 1–5 and 30-minute Delayed Recall trial;^81^ (3) non-verbal memory measured with the Rey-Osterrieth Complex Figure Test (ROCFT) – the Recall condition after 3 minutes;^82^ (4) visuospatial function measured with the ROCFT – the Copy condition^82^ and the Clock Drawing Test;^83^ (5) executive function measured with the Trail Making Test (TMT) B, the Controlled Oral Word Association Test (Czech version with letters N, K, and P),^80^ and the Prague Stroop Test, a color-word condition;^84^ (6) attention and working memory measured with the Forward and Backward Digit Spans and the TMT A;^80^ and (7) language measured with the Boston Naming Test, a 30 odd-items version and the Category Fluency Test – Animals and Vegetables.^80^ The maximum time to complete the TMT A and B was 180 s and 300 s, respectively, and those who were unable to complete it in a given time were scored as 181 s and 301 s, respectively. The self-report Geriatric Depression Scale, a 15-item version (GDS-15),^85^ and the Beck Anxiety Inventory (BAI)^86^ were administered to evaluate depressive and anxiety symptoms. The cognitive characteristics of all study participants and the participants from the memory clinic cohort with biomarkers stratified by amyloid-β status are shown in Tables 1 and 2, respectively.

#### CSF analysis of AD biomarkers

CSF samples were obtained by lumbar puncture in the supine position, collected in 8-mL polypropylene tubes, combined, gently mixed, centrifuged, divided into aliquots, and stored at –80°C until analysis. Stored CSF samples were thawed and vortexed before biomarker analysis. CSF collection, processing and archiving was performed in accordance with European recommendations.^87^ CSF amyloid-β_1-42_, p-tau_181_ and total tau were analyzed using commercial ELISA (Euroimmun, Lübeck, Germany) in the Cerebrospinal Fluid Laboratory, Institute of Immunology and Department of Neurology, Second Faculty of Medicine, Charles University and Motol University Hospital. Unbiased cut-offs of less than 665 pg/mL and more than 48 pg/mL and 358 pg/mL were used to define amyloid-β_1-42_, p-tau_181_ and total tau positivity, respectively.^23^^,^^88^

#### Amyloid PET imaging

Dual-phase amyloid PET was used to assess amyloid-β positivity and regional brain hypoperfusion as a marker of neurodegeneration. PET images were acquired using a Biograph 40 TrueV HD PET/CT scanner (Siemens Healthineers AG, Erlangen, Germany). The participants received a single intravenous dose of ^18^F-flutemetamol (Vizamyl, GE Healthcare, Chicago, IL) with a gross activity of 206.7 ± 12.7 MBq. Non-contrast low-dose CT brain images were acquired for attenuation correction prior to the PET scans. A PET list-mode acquisition was performed in two phases: early (perfusion) and late (amyloid-β). The early-phase images were acquired at the time of ^18^F-flutemetamol administration for 8 minutes rebinned into dynamic datasets of 2 x 4 min for motion checking. They were iteratively reconstructed to a 168 x 168 matrices with three iterations, after attenuation, scatter, and point spread function correction. The late-phase images were acquired 90 min after ^18^F-flutemetamol administration for 10 minutes and iteratively reconstructed to a 128 x 128 matrix with other parameters as described above, including rebinning into dynamic sequences for motion checking.^89^ ^18^F-flutemetamol PET images were visually read (as positive or negative) by a certified nuclear medicine specialist. The early-phase images were evaluated for perfusion deficits in the gray matter using the "Warm Metal" isocontour color scale. The late-phase images were evaluated for specific amyloid-β uptake in the gray matter using the GM-EDGE method that visualizes the gray-white matter borders derived from the early-phase images.^89^ Eight specific brain regions were assessed, including the frontal lobe, lateral temporal lobe, anterior cingulate, posterior cingulate, precuneus, temporoparietal area, insula, and striatum. If any of these regions was abnormal, the finding was classified as positive for amyloid-β.

#### Magnetic resonance imaging

##### Acquisition

MRI images were acquired on a Siemens Avanto 1.5T scanner (Siemens AG, Erlangen, Germany) with a 12-channel phased-array head coil using high-resolution three-dimensional T1-weighted (3D T1w) Magnetization-Prepared Rapid Gradient Echo (MPRAGE) sequence with the following parameters: TR/TE/TI = 2,000/3.08/1,100 ms, flip angle = 15°, 192 continuous partitions, slice thickness = 1.0 mm, and in-plane resolution = 1 mm.^90^ All images were visually inspected by a neuroradiologist to exclude participants with tumors, cortical infarcts, hydrocephalus, or other major brain pathology, and by a trained data analyst for quality control such as excessive motion artifacts. The 3D T1w images of a sufficient technical quality were available for 248 participants, including CN (n = 39), SCD (n = 72), aMCI (n = 113), and mild dementia (n = 24) participants.

##### Hippocampal and entorhinal cortex segmentation

We used an image analysis protocol from our previous studies.^23^^,^^91^^,^^92^ Specifically, a previously published processing pipeline based on a CBAS template was used to measure volumes of the hippocampal head, body and tail, volumes of the anterolateral and posteromedial entorhinal cortex, and estimated total intracranial volume (eTIV).^23^^,^^92^ The skull-stripped 3D T1w images corrected for magnetic field (B1) inhomogeneity using the N4 algorithm were processed using statistical parametric mapping (SPM8, Wellcome Trust Center for Neuroimaging)^93^ and the VBM8-toolbox (http://dbm.neuro.uni-jena.de/vbm/) implemented in MatLab R2015b (MathWorks, Natick, MA). We used a previously published CBAS template based on manual segmentation of the hippocampal and entorhinal cortex subregions aligned in MNI space, derived from 26 cognitively normal older adults recruited from the CBAS.^69^ The CBAS template was registered and diffeomorphically warped into individual participants’ space using Advanced Normalization Tools package (http://stnava.github.io/ANTs/) with a cross-correlation method, 100 x 100 x 50 iterations, and symmetric normalization applied on a 0.25 threshold. The resulting warp field was used to transform ROI masks of individual hippocampal and entorhinal cortex subregions into the participants’ space. The ROIs masks were subsequently masked with a gray matter ROI and their volumes were extracted. Volumes of the hippocampal body and tail were summed into posterior hippocampal volume. To reduce the number of multiple comparisons, only volumes of the posterior subregions of the hippocampus and entorhinal cortex (i.e., the posterior hippocampus and posteromedial entorhinal cortex) that are most closely associated with spatial navigation^14^^,^^15^ were used in the statistical analyses.

#### Protocol for creating the CBAS template

A population-based template was created using structural 3D T1w images of 26 CN older adults recruited from the CBAS.^69^ The 3D T1w images were processed using the ANTs package.^94^ First, an initial registration template was created, and then we proceeded to create the final population template by iteratively registering 3D T1w images into the initial template. Manual segmentation of the hippocampus and entorhinal cortex was performed for each participant used to create the population-based template. The hippocampus and entorhinal cortex were delineated manually in the coronal plane using anatomical landmarks according to the previously published manual segmentation protocol.^95^ The hippocampus was divided into three subregions – the head, the body, and the tail, and the entorhinal cortex was divided into the anterolateral and posteromedial subregions according to the previously published segmentation protocols,^95^^,^^96^ respectively. Manually delineated ROIs were normalized to MNI space using deformation fields obtained during the template creation. A template for each structure (i.e., the hippocampal head, body, and tail, anterolateral and posteromedial entorhinal cortex) was created using the same procedure as the initial template creation.^92^ Individual templates were split into the left and right masks using the left and right hemispheric ROIs. Resulting masks were rescaled into values 0–100 to represent probabilistic distribution.

#### Cortical segmentation

The FreeSurfer image analysis suite (v7.4.0; http://surfer.nmr.mgh.harvard.edu/) was used to measure thickness of the right and left precuneus, isthmus cingulate, posterior cingulate, superior parietal gyrus, inferior parietal gyrus, and supramarginal gyrus based on the designation in the Desikan-Killiany atlas.^97^ Thickness of the posterior parietal cortex, a composite region, was computed as the area-weighted mean thickness of the three latter regions. Similarly, the thickness of the retrosplenial cortex, a fused region, was derived as the area weighted mean thickness of the ventral portions of the isthmus cingulate and posterior cingulate regions from the Desikan Killiany atlas based on previous functional^68^^,^^98^ and anatomical studies^99^ of the retrosplenial cortex.

#### Basal forebrain segmentation

We used the same preprocessing procedures as described above (i.e., skull stripping and B1 inhomogeneity correction) and followed the previously published protocol to measure volume of the basal forebrain.^100^^,^^101^^,^^102^ MRI data were processed using SPM8 and the VBM8-toolbox implemented in MatLab R2015b. As in our previous studies,^90^^,^^91^^,^^92^ we used a mask of the basal forebrain based on a cytoarchitectonic map of the basal forebrain cholinergic nuclei aligned in MNI space, derived from combined histology and MRI of the postmortem brain. Location of the basal forebrain nuclei was identified using histological staining, manually transferred into postmortem MRI space and subsequently transformed into MNI standard space.^100^^,^^103^ We nonlinearly registered all the images into the MNI152 template and used the resulting DARTEL parameters^104^ to warp the cytoarchitectonic map into individual brain scans. Volumes of the right and left basal forebrain were extracted and averaged across both hemispheres. All volumes were normalized to eTIV using the previously published regression formula.^76^^,^^105^ The outputs were visually inspected for image and segmentation quality by an experienced reader blinded to clinical and biomarker data. The MRI characteristics of all study participants and the participants from the memory clinic cohort with biomarkers stratified by amyloid-β status are shown in Tables 1 and 2, respectively.

### Quantification and statistical analysis

#### Statistical analysis

All analyses were performed in SPSS, version 28.0, (IBM, Armonk, NY, USA). The R software (R Foundation for Statistical Computing, Vienna, Austria; https://www.rproject.org) was used to generate violin plots and correlation heatmaps. Statistical significance was set at two-tailed p < .05. The descriptive characteristics are shown as means and SDs for continuous variables and proportions for categorical variables. All continuous variables were standardized to z-scores to allow comparisons across different measures. Data with non-normal distribution (i.e., the SSNCQ-s, SSNCQ-i and QuENA-i scores, spatial navigation measures, and CSF biomarker levels) were log-transformed before z-transformation. Missing data from administered questionnaires and spatial navigation tasks (< 10%) were imputed using multiple imputation with an iterative Markov chain Monte Carlo method,^106^ which generated five imputed datasets that were pooled into the final dataset to reduce bias in data estimates. Cronbach’s alpha was used to assess the internal consistency of the questionnaires. For statistical analysis, the entire memory clinic cohort and each clinical group were stratified according to amyloid-β status into A-negative and A-positive. Eight AT(N) biomarker profiles were stratified into three biomarker groups based on the number of positive A and T biomarkers: A and T negative (A-T-), A or T positive (A+T-/A-T+), and A and T positive (A+T+).

Group differences in demographics, cognitive performance, life-space mobility, volumes/thicknesses of AD-related brain regions, and AD biomarkers were analyzed with one-way analysis of variance for continuous variables and χ^2^ tests for categorical variables. The clinically and biomarker-defined participants with (n = 187) and without (n = 75) completed informant versions of the questionnaires were also compared. Differences in questionnaire scores and spatial navigation performance were analyzed using a series of general linear models and linear mixed models with random intercepts and slopes, and navigation trials as a repeated measure, respectively, adjusted for age, gender, education, and life-space mobility (the LSA score) to account for differences in demographics. All post-hoc tests were adjusted for multiple comparisons using false discovery rate (FDR) correction. Receiver operating characteristic (ROC) analysis was used to assess the accuracy of the questionnaires to discriminate between the groups. Areas under the curves (AUC) with 95% CIs are reported.

In the memory clinic cohort including participants with SCD, aMCI and mild dementia, the associations of questionnaire scores and spatial navigation performance with demographics, volumes/thicknesses of AD-related brain regions, AD biomarkers (i.e., CSF amyloid-β_1-42_, CSF p-tau_181_ and CSF total tau levels, and amyloid PET negative vs. amyloid PET positive), and biomarker status (i.e., A-negative vs. A-positive, A-T- vs. A-T+/A+T-, A-T- vs. A+T+, and A-T+/A+T- vs. A+T+) were assessed using Pearson correlation (r) including point-biserial correlation for dichotomous variables. Associations between questionnaire scores and spatial navigation performance were then assessed using a series of linear mixed models with random intercepts and slopes, and navigation trials as a repeated measure, adjusted for age, gender, education, and life-space mobility. Interactions of questionnaire scores with group status adjusted for age, gender, education, and life-space mobility were tested to assess the modifying effect of the group. If the interaction was significant (p < .05; unadjusted), separate linear mixed models were performed for each group. Associations of volumes/thicknesses of AD-related brain regions, AD biomarkers, and biomarker status with questionnaire scores were then assessed using a series of linear regression models adjusted for age, gender, education, and life-space mobility. Results are shown as regression coefficients (β) with 95% CIs. FDR correction was used to adjust for multiple comparisons. Adjusted and unadjusted statistical significance is reported. A series of path analyses adjusted for age, gender, education, and life-space mobility was used to assess the mediating effect of spatial navigation performance in the associations between volumes/thicknesses of AD-related brain regions and questionnaire scores (Figure 4). The path analysis included only variables for which biologically plausible significant associations were found in previous analyses. The bootstrapping method was used to test for the significance of the mediating effect, and β with 95% CIs are reported.

## Supplemental information

Document S1. Figures S1–S5

Table S1. Demographic, cognitive and neuroimaging data in participants with and without missing informant-reported spatial navigation questionnaires Values are mean (SD) except for gender. a Based on a sample with complete brain imaging data (n = 248) with CN (n = 39), SCD (n = 72), aMCI (n = 113) and mild dementia (n = 24) participants. b Normalized to estimated total intracranial volume. Key: CN, cognitively normal; SCD, subjective cognitive decline; aMCI, amnestic mild cognitive impairment; LSA, Aging Life-Space Assessment; MMSE, Mini-Mental State Examination; GDS-15, Geriatric Depression Scale 15-item version; BAI, Beck Anxiety Inventory; LM, Logical Memory; RAVLT, Rey Auditory Verbal Learning Test; RAVLT 1–5, trials 1 to 5 total; RAVLT 30, delayed word recall after 30 min; TMT A and B, Trail Making Tests A and B; COWAT, Controlled Oral Word Association Test (Czech version with letters N, K and P); ROCFT-C, Rey-Osterrieth Complex Figure Test – the Copy condition; ROCFT-R, Rey-Osterrieth Complex Figure Test – the Recall condition after 3 min; DSF, Digit Span Forward total score; DSB, Digit Span Backward total score; CDT, Clock Drawing Test – Cohen’s scoring; SVF, Semantic Verbal Fluency; BNT, Boston Naming Test (30-item version); PST, Prague Stroop Test – colors; pmEC, posteromedial entorhinal cortex; BF, basal forebrain.

Table S2. Demographic, cognitive and neuroimaging data in biomarker-defined memory clinic cohort participants with and without missing informant-reported spatial navigation questionnaires Values are mean (SD) except for gender and amyloid PET positivity. a Based on a sample with complete brain imaging data (n = 129) with SCD amyloid negative (n = 33), SCD amyloid positive (n = 9), aMCI amyloid negative (n = 33), aMCI amyloid positive (n = 45) and mild dementia amyloid positive (n = 9) participants. b Normalized to estimated total intracranial volume. c Based on a sample with CSF data (n = 66) with SCD amyloid negative (n = 7), SCD amyloid positive (n = 3), aMCI amyloid negative (n = 17), aMCI amyloid positive (n = 30) and mild dementia amyloid positive (n = 9) participants. d Based on a sample with amyloid PET data (n = 100) with SCD (n = 38), aMCI (n = 56) and mild dementia amyloid positive (n = 6) participants. Key: SCD, subjective cognitive decline; aMCI, amnestic mild cognitive impairment; LSA, Aging Life-Space Assessment; MMSE, Mini-Mental State Examination; GDS-15, Geriatric Depression Scale 15-item version; BAI, Beck Anxiety Inventory; LM, Logical Memory; RAVLT, Rey Auditory Verbal Learning Test; RAVLT 1–5, trials 1 to 5 total; RAVLT 30, delayed word recall after 30 min; TMT A and B, Trail Making Tests A and B; COWAT, Controlled Oral Word Association Test (Czech version with letters N, K and P); ROCFT-C, Rey-Osterrieth Complex Figure Test – the Copy condition; ROCFT-R, Rey-Osterrieth Complex Figure Test – the Recall condition after 3 min; DSF, Digit Span Forward total score; DSB, Digit Span Backward total score; CDT, Clock Drawing Test – Cohen’s scoring; SVF, Semantic Verbal Fluency; BNT, Boston Naming Test (30-item version); PST, Prague Stroop Test – colors; pmEC, posteromedial entorhinal cortex; BF, basal forebrain; CSF, cerebrospinal fluid.
