# Supplementary material for: Spatial navigation questionnaires as a supportive diagnostic tool in early Alzheimer’s disease
Source: iScience. 2024 Apr 26;27(6):109832. doi: 10.1016/j.isci.2024.109832 (PMC11108981; doi:10.1016/j.isci.2024.109832)
Supplement: Document S1. Figures S1–S5 [file mmc1.pdf]

iScience, Volume *27*

Supplemental information

Spatial navigation questionnaires as a supportive diagnostic tool in early Alzheimer's disease

Martina Laczó, Radka Svatkova, Ondrej Lerch, Lukas Martinkovic, Terezie Zuntychova, Zuzana Nedelska, Hana Horakova, Martin Vyhnalek, Jakub Hort, and Jan Laczó

Supplementary Figures

Figure S1. Correlations between spatial navigation performance, demographics, regional brain measures, AD biomarkers, and biomarker status

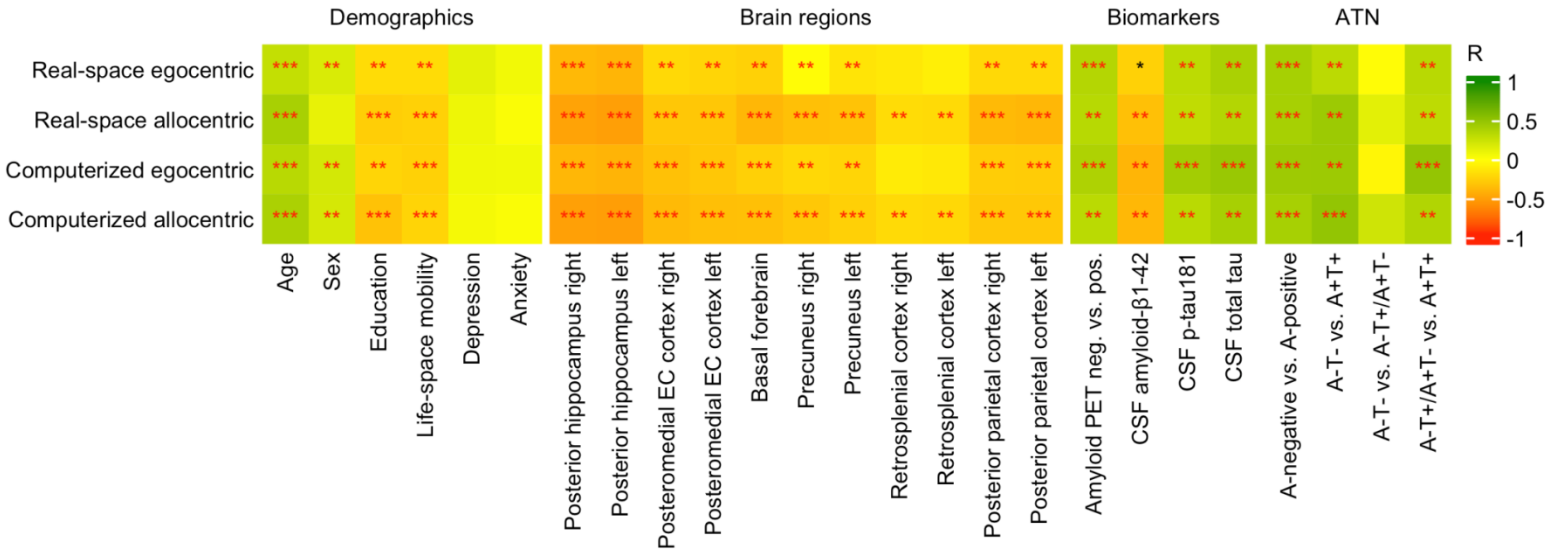

Notes: *p <.05; ** p <.01; and *** p <.001. Red asterisks indicate significant correlations after adjustment for multiple comparisons using false discovery rate (FDR) correction.

Key: egocentric, egocentric navigation task; allocentric, allocentric navigation task; ATN, ATN biomarker status; EC, entorhinal cortex; CSF, cerebrospinal fluid; A-negative, amyloid-β negative; A-positive, amyloid-β positive; A, amyloid-β; T, tau; -, normal; +, abnormal.

Figure S2. Subjective Spatial Navigation Complaints Questionnaire (self-reported questionnaire, SSNCQ-s)

**I have had difficulties in the last 3 months with:**					
Orientation in my home					
	never	less than once a week	approximately once a week	several times a week	every day
Orientation in my neighborhood					
	never	less than once a week	approximately once a week	several times a week	every day
Orientation in my town					
	never	less than once a week	approximately once a week	several times a week	every day
Orientation outside of my town					
	never	less than once a week	approximately once a week	several times a week	every day
**I have been lost in the last 3 months:**					
in my flat					
	never	less than once a week	approximately once a week	several times a week	every day
in my neighborhood					
	never	less than once a week	approximately once a week	several times a week	every day
in my town					
	never	less than once a week	approximately once a week	several times a week	every day
in the other town, than where I live					
	never	less than once a week	approximately once a week	several times a week	every day
**With respect to places that I visit every day or almost every days, in the last 3 months, my ability to orient myself has been _______ compared to when I was young:**					
	same or better	little worse	much worse	significantly worse	
**With respect to places that I visit several times a year, in the last 3 months, my ability to orient myself has been _______ compared to when I was young:**					
	same or better	little worse	much worse	significantly worse	
**In the last 3 months, I have had to ask for directions more often than in the past:**					
	never	less than once a week	approximately once a week	several times a week	every day
**In the last 3 months, I have had difficulties getting oriented in my supermarket:**					
	never	less than once a week	approximately once a week	several times a week	every day
**Because of worries that I may get lost, I have had to:**					
	reduce traveling out of my town.			yes	no
	reduce traveling to my relatives and friends.			yes	no
	reduce activities around my home (shopping, go to post, *etc.*).			yes	no

Figure S3. Santa Barbara Sense of Direction Scale (self-reported questionnaire, SBSOD-s)

SANTA BARBARA SENSE-OF-DIRECTION SCALE Participant: ____________

The following statements ask you about your spatial and navigational abilities, preferences, an[illegible] experiences. After each statement, you should circle a number to indicate your level of agreement with the statement. Circle "1" if you strongly agree that the statement applies to yo[illegible] "7" if you strongly disagree, or some number in between if your agreement is intermediate. Circle "4" if you neither agree nor disagree.

1. I am very good at giving directions.
strongly agree 1 2 3 4 5 6 7 strongly disagree

2. I have a poor memory for where I left things.
strongly agree 1 2 3 4 5 6 7 strongly disagree

3. I am very good at judging distances.
strongly agree 1 2 3 4 5 6 7 strongly disagree

4. My "sense of direction" is very good.
strongly agree 1 2 3 4 5 6 7 strongly disagree

5. I tend to think of my environment in terms of cardinal directions (N, S, E, W).
strongly agree 1 2 3 4 5 6 7 strongly disagree

6. I very easily get lost in a new city.
strongly agree 1 2 3 4 5 6 7 strongly disagree

7. I enjoy reading maps.
strongly agree 1 2 3 4 5 6 7 strongly disagree

8. I have trouble understanding directions.
strongly agree 1 2 3 4 5 6 7 strongly disagree

9. I am very good at reading maps.
strongly agree 1 2 3 4 5 6 7 strongly disagree

10. I don't remember routes very well while riding as a passenger in a car.
strongly agree 1 2 3 4 5 6 7 strongly disagree

11. I don't enjoy giving directions.
strongly agree 1 2 3 4 5 6 7 strongly disagree

12. It's not important to me to know where I am.
strongly agree 1 2 3 4 5 6 7 strongly disagree

13. I usually let someone else do the navigational planning for long trips.
strongly agree 1 2 3 4 5 6 7 strongly disagree

14. I can usually remember a new route after I have traveled it only once.
strongly agree 1 2 3 4 5 6 7 strongly disagree

15. I don't have a very good "mental map" of my environment.
strongly agree 1 2 3 4 5 6 7 strongly disagree

Figure S4. Questionnaire on Everyday Navigational Ability (self-reported questionnaire, QuENA-s)

QuENA, Patient Version
Please select WHERE the symptom(s) would occur.
0: Never; Those symptom(s) has never occurred
1: Less familiar; I have ever been there but seldom visit there.
2: Fairly familiar; I go there regularly.
3: Very familiar; I go there almost every day.

1. Have you ever failed to recognize a landmark?
0: Never
1: Yes, but only in less familiar place
2: Yes, in places which I visit fairly regularly
3: Yes, in places very familiar to me

2. Have you ever failed to recognize street scenes?
0: Never
1: Yes, but only in less familiar place
2: Yes, in places which I visit fairly regularly
3: Yes, in places very familiar to me

3. Have you ever lost the sense of familiarity?
0: Never
1: Yes, but only in less familiar place
2: Yes, in places which I visits fairly regularly
3: Yes, in places very familiar to me

4. Have you ever been unable to describe the route between your home and a destination?
0: Never
1: Yes, but only in less familiar place
2: Yes, in places which I visit fairly regularly
3: Yes, in places very familiar to me

5. Have you ever made a wrong turn at a crossroad?
0: Never
1: Yes, but only at less familiar crossroads
2: Yes, at crossroads which I know fairly well
3: Yes, at crossroad which I know very well

6. Have you ever accidently forgotten to make a turn due to inattention?
0: Never
1: Yes, but only in less familiar place
2: Yes, in places which I visit fairly regularly
3: Yes, occurred in the place very familiar to me (such as passing by my home)

7. Have you ever accidently ended up in an unfamiliar place due to inattention?
0: Never
1: Yes, but only at less familiar crossroads
2: Yes, at crossroads which I know fairly well
3: Yes, at crossroad which I know very well

8. When you were at the start point (eg. at home), have you ever failed to point out the direction of a destination?
0: Never
1: Yes, but only less familiar destinations
2: Yes, for destinations which I visit fairly regularly
3: Yes, for very familiar destinations (such as my home or neighborhood)

9. When you were on the road, have you ever failed to point out the direction of your home or destination?
0: Never
1: Yes, but only in less familiar place
2: Yes, in places which I visit fairly regularly
3: Yes, in places very familiar to me

10. When you were indoors, have you ever failed to point out the exit direction or where the bathroom is?
0: Never
1: Yes, but in less familiar buildings (such as a department store)
2: Yes, in buildings which I visited fairly regularly (such as a hospital)
3: Yes, in buildings very familiar to me (such as my house)

Figure S5. Life-Space Assessment (LSA)

LIFE-SPACE ASSESSMENT

The following questions refer to your activities just within the past month.

LIFE-SPACE LEVEL			FREQUENCY				INDEPENDENCE	SCORE
**During the past four weeks, have you been to . . .**			**How often did you get there?**				**Did you use aids or equipment? Did you need help from another person?**	Level X Frequency X Independence
Life-Space Level 1. . . **Other rooms of your home besides the room where you sleep?**	Yes<br>1	No<br>0	Less than 1 /week<br>1	1-3 times /week<br>2	4-6 times /week<br>3	Daily<br>4	1 = personal assistance<br>1.5 = equipment only<br>2 = no equipment or personal assistance	
Score	____(LS1) X		____(LS1F)			X	____(LS1I) =	*Level 1 Score*
Life-Space Level 2. . . **An area outside your home such as your porch, deck or patio, hallway (of an apartment building) or garage, in your own yard or driveway?**	Yes<br>2	No<br>0	Less than 1 /week<br>1	1-3 times /week<br>2	4-6 times /week<br>3	Daily<br>4	1 = Personal assistance<br>1.5 = Equipment only<br>2 = No equipment or personal assistance	
Score	____(LS2) X		____(LS2F)			X	____(LS2I) =	*Level 2 Score*
Life-Space Level 3. . . **Places in your neighborhood, other than your own yard or apartment building?**	Yes<br>3	No<br>0	Less than 1 /week<br>1	1-3 times /week<br>2	4-6 times /week<br>3	Daily<br>4	1 = Personal assistance<br>1.5 = Equipment only<br>2 = No equipment or personal assistance	
Score	____(LS3) X		____(LS3F)			X	____(LS3I) =	*Level 3 Score*
Life-Space Level 4. . . **Places outside your neighborhood, but within your town?**	Yes<br>4	No<br>0	Less than 1 /week<br>1	1-3 times /week<br>2	4-6 times /week<br>3	Daily<br>4	1 = Personal assistance<br>1.5 = Equipment only<br>2 = No equipment or personal assistance	
Score	____(LS4) X		____(LS4F)			X	____(LS4I) =	*Level 4 Score*
Life-Space Level 5. . . **Places outside your town?**	Yes<br>5	No<br>0	Less than 1 /week<br>1	1-3 times /week<br>2	4-6 times /week<br>3	Daily<br>4	1 = Personal assistance<br>1.5 = Equipment only<br>2 = No equipment or personal assistance	
Score	____(LS5) X		____(LS5F)			X	____(LS5I) =	*Level 5 Score*
							**TOTAL SCORE (ADD)**	*Sum of Levels*
